# Isolated, neglected, and likely threatened: a new species of *Magoniella* (Polygonaceae) from the seasonally dry tropical forests of Northern Colombia and Venezuela revealed from nuclear, plastid, and morphological data

**DOI:** 10.3389/fpls.2024.1253260

**Published:** 2024-07-23

**Authors:** José Aguilar-Cano, Oscar Alejandro Pérez-Escobar, Camila Pizano, Eduardo Tovar, Alexandre Antonelli

**Affiliations:** ^1^ Herbario JBB, Jardín Botánico de Bogotá, Bogotá, Colombia; ^2^ Royal Botanic Gardens, Kew, Richmond, United Kingdom; ^3^ Herbario Icesi, Departamento de Biología, Universidad Icesi, Cali, Colombia; ^4^ Laboratorio de Genética de la Conservación, Instituto de Investigaciones de Recursos Biológicos Alexander von Humboldt, Bogotá, Colombia; ^5^ Gothenburg Global Biodiversity Centre, Department of Biological and Environmental Sciences, University of Gothenburg, Gothenburg, Sweden; ^6^ Department of Biology, University of Oxford, Oxford, United Kingdom

**Keywords:** dry tropical forests, systematics, molecular diagnosis, phylogenetics, Triplarideae, taxonomy, deforestation, extinction risk

## Abstract

Seasonally tropical dry forests (SDTFs) in the American tropics are a highly diverse yet poorly understood and endangered ecosystem scattered from Northern Mexico to Southern Argentina. One floristic element of the STDFs is the genus *Magoniella* (Polygonaceae), which includes two liana species, *M. laurifolia* and *M. obidensis*, which have winged fruits and are distributed from Costa Rica to Southern Brazil. In a field expedition to the SDTFs of the Colombian Caribbean in 2015, morphologically distinctive individuals of *Magoniella* were found. In this study, we investigated the species boundaries within *Magoniella* and determined the phylogenetic position of these morphologically distinctive individuals in the tribe Triplaridae. We compiled morphological trait data across 19 specimens of both species and produced newly sequenced nuclear–plastid DNA data for *M. obidensis*. Morphometric analyses revealed significant differences in fruit length and perianth size among individuals from the Colombian Caribbean compared to *M. obidensis* and bract length when compared to *M. laurifolia*. Maximum likelihood analysis of non-conflicting nuclear and plastid datasets placed the Colombian Caribbean individuals as sister to *M. obidensis* with maximum statistical support. Additionally, pairwise sequence comparisons of the nuclear ribosomal ITS and the *lfy2i* loci consistently showed 15-point mutations (10 transitions, five transversions) and six 2 bp-long substitutions that differ between *M. obidensis* and the Colombian Caribbean individuals. Our morphological and molecular evidence thus suggests that the Colombian Caribbean individuals of *Magoniella* represent a divergent population from *M. laurifolia* and *M. obidensis*, which we describe and illustrate as a new species, *M. chersina*. Additionally, we provide nomenclatural updates for *M. laurifolia* and *M. obidensis*. This study highlights the power of combining morphological and molecular evidence in documenting and naming plant diversity.

## Introduction

In the American tropics (the Neotropics), seasonally tropical dry forests (SDTFs) constitute a diverse yet disjunct biome, found in fragmented areas from Mexico in the North to Argentina in the South (Pennington et al., 2009; Linares-Palomino et al., 2011). This biome is characterised by a mean annual temperature above 17°C (Holdridge, 1947) and annual precipitation below ~1800 mm, with a dry season lasting 3–6 months, during which the monthly precipitation is less than 100 mm (Banda-R et al., 2016). The vegetation in SDTFs is deciduous or semi-deciduous during the dry season. Many trees and shrubs shed their leaves, and herbs usually survive as seeds or underground. This biome can be distinguished from more open, grass-rich savannas by its closed canopy during the wet season, as well as the presence of cacti and other succulents. These are not well adapted to recurrent fires characteristic of savannas, and there are fewer grasses (Pennington et al., 2010; Banda-R et al., 2016).

Compared to the two other major lowland neotropical biomes—rainforests and savannas—SDTFs exhibit distinctive phylogenetic and biogeographic patterns (Pennington and Lavin, 2016). Species found in STDF fragments usually have a longer evolutionary persistence, resulting in narrower distribution ranges due to limited dispersal or eco-physiological barriers, preventing their range expansions. These fragmented SDTF areas are often referred to as “enclaves” or “nuclei” (Pennington et al., 2009). Based on environmental and floristic characteristics, the Caribbean coast of Colombia and Venezuela’s STDF nuclei are treated as a single area of dry forest, distinct from the adjacent northern inter-Andean valleys of Colombia and Venezuela (Pennington et al., 2004; Linares-Palomino et al., 2011; Banda-R et al., 2016; Dinerstein et al., 2017; González-M et al., 2018; Pérez-Escobar et al., 2022). Banda-R et al. (2016) categorized both nuclei in the “higher level northern group,” yet their somewhat disjunct distribution suggests that the Amazonian–Chocoan rainforests have served as a barrier for biotic exchange between these SDTF nuclei (Pérez-Escobar et al., 2019).

Among the numerous Neotropical plants with extensive distributions that are characteristic of certain biomes, the genus M*agoniella* Adr.Sanchez (Polygonaceae) stands out. It was erected to encompass species previously classified under the genus *Ruprechtia* C.A.Mey., based on morphological and phylogenetic evidence presented in a study focussed on the tribe Triplarideae (Sanchez and Kron, 2011). This tribe currently comprises three additional genera: *Triplaris* Loefl., *Ruprechtia* and *Salta* Adr.Sanchez. Collectively, these four genera include 53 species (Govaerts et al., 2021; POWO, 2023) of dioecious trees, shrubs and lianas, all characterised by stipules (ochreas) protecting the stem and leaf buds, three-winged fruits, flowers with six tepals and six stamens, and three-lobed or simple seeds.

Each of these genera displays distinct preferences within lowland neotropical biomes. For instance, *Salta triflora* (Griseb.) Adr.Sanchez, the sole species in its genus, occurs mainly in forests and shrublands in the Chaco savannas, occasionally appearing in seasonally flooded forests (Pendry, 2004). In contrast, most *Triplaris* species are found in seasonally flooded forests in Amazonia, along rivers and open areas, while those outside of Amazonia are located in patches of dry forests in the Andean region (Brandbyge, 1986; Sanchez and Kron, 2011). Similarly, *Ruprechtia* species are distributed in various SDTF regions, with some restricted to particular dry forest enclaves, while others have a wide distribution in the humid rainforest of Central and South America (Pendry, 2004). *Magoniella obidensis* (Huber) Adr.Sanchez and *M. lauriflora* (Cham. & Schltdl.) Adr.Sanchez, the only two recognised species in the genus (Pendry, 2004; Sanchez and Kron, 2011), are found in the tropical rainforests of the Amazon basin and the Atlantic Forest, respectively. The genus differs from its sister genera in the Triplarideae by its liana habit, hollow stems, and large green fruits with red sepals (Sanchez and Kron, 2011).

During floristic exploratory expeditions conducted in the Colombian Caribbean region between 2015 and 2016, a specimen of an unusual Polygonaceae plant was collected (Aguilar-Cano et al., 2016). Its partial infructescence featuring two fruits, subtended by a single tubular bracteole, suggested a morphological similarity with *Ruprechtia*. However, its strict liana habit and large green fruits with red sepals corresponded to diagnostic traits of *Magoniella*. Two additional specimens found in the K and MO herbaria (Thiers, 2021, continuously updated) had also been collected from two other localities in the tropical dry forest of the Caribbean coast of Colombia and Venezuela. These specimens had been overlooked as a distinct species by taxonomists for nearly 100 years but displayed morphological affinities with the *Magoniella* specimen collected by Aguilar-Cano (Aguilar-Cano et al., 2016).

In this study, we compiled morphological and molecular phylogenetic evidence to propose a new species of *Magoniella* to science and to ascertain its phylogenetic position within the tribe Triplaridae. To achieve this, we produced nuclear and plastid DNA sequences and collected data on morphological traits to investigate species boundaries. We employed an integrative taxonomic approach, using operational criteria to assess lineage separation, integrate multiple lines of evidence, and document biodiversity (De Queiroz, 2007; Freudenstein et al., 2016; Grace et al., 2021; Wells et al., 2021). Specifically, we sampled two populations of the putative new species, included 198 sequences from 33 species (representing the entire generic diversity of Triplarideae), and assessed their morphological variation and distribution ranges. As a result, we unequivocally placed the potential new species in the genus *Magoniella* and confirmed that these collections from the Colombian and Venezuelan Caribbean dry forests indeed represent a distinct species, *Magoniella chersina*.

## Materials and methods

### DNA extraction, amplification, and sequencing

DNA extraction from leaf tissue samples was carried out using the CTAB protocol from Ivanova et al. (2008). Two plastid markers, the *ndhF* coding region and the *rps16-trnK* noncoding spacers, and two nuclear regions, the internal transcribed spacer (ITS) and the second intron of the low-copy nuclear region *Leafy* (*lfy*2*i*), were amplified and sequenced using the primer combinations reported in **Supplementary Table S1**. A total of 20 new sequences were generated in this study (voucher information provided in **Supplementary Table S2**). Amplification of selected regions was performed in a final volume of 15 μL containing the following: 2 μL of template DNA (~10–50 ng), 1X Taq buffer ((NH4) 2SO4), 200 μM of each deoxynucleoside triphosphate, 2 mM MgCl2, 0.2 μM of each primer, 0.4 μg/μL bovine serum albumin, and 1 U of Taq DNA polymerase. The PCR cycling conditions consisted of a first cycle of denaturation at 94°C for 3 min; followed by 35 cycles of 94°C for 30 s, TA (50°C *rps16-trnk*, ndhF, ITS; 56°C lfy2i) for 40 s, and 72°C for 2 min; and a final extension cycle at 72°C for 5 min. The sequences obtained were first assembled and edited using Geneious software version 10.26 (http://www.geneious.com, Kearse et al., 2012).

### Phylogenetic analysis

Our newly generated sequences were combined with the DNA dataset of Sanchez and Kron (2011), which targeted four plastid (*mat*K, *ndh*F, *trn*V-*ndh*C, and *rps*16-*trn*K) and two nuclear (ITS, *lfy2i*) DNA regions and represented 62% of known Triplarideae species. The combined DNA dataset sampled 33 putative species: nine from *Triplaris* (of the 19 known to science [POWO, 2023]), 17 from *Ruprechtia* (of the 31 [POWO, 2023]), one from the monotypic genus *Salta*, and two from *Magoniella*, as well as four outgroup species in family Polygonaceae used by Sanchez and Kron (2011): *Antigonon leptopus* Hook. & Arn., *Coccoloba swartzii* Meisn., *Eriogonum alatum* Torr., and *Gymnopodium floribundum* Rolfe. Species names, voucher specimens (for new sequences generated by this study), and all GenBank accession numbers of sequences included in phylogenetic analyses are listed in **Supplementary Table S2**.

Multiple sequence DNA alignments were conducted with the software MAFFT v. 7.453 (Katoh et al., 2002), using a maximum of 1,000 refinement iterations (flag*—maxiter*), and the option *globalpair* (i.e., G-INS-s high accuracy strategy). Maximum likelihood (ML) analyses were performed using the software RAXML-HPC v.8.2.4 (Stamatakis, 2014), using the general time-reversible nucleotide substitution model, a gamma distribution model (20 categories) and 500 non-parametric bootstraps (MLBS) as implemented in the CIPRES Science Gateway computing facility (Miller et al., 2015) (https://www.phylo.org/). Three ML phylogenetic analyses were carried out from three datasets: (*i*) combined plastid dataset, (*ii*) combined nuclear dataset, and (*iii*) total non-conflicting combined nuclear and plastid dataset following an analysis on topological discordance between both datasets [i.e., Pérez-Escobar et al. (2016, 2017)], using the software PACo (Balbuena et al., 2013) (http://www.uv.es/cophylpaco/) as implemented in R.

### Morphological characters and geographical distribution

Species delimitation in *Magoniella* has predominantly relied on the morphological variation observed in fruits, because variability in vegetative and male reproductive characters is minimal (Pendry, 2004). Specimens of *Magoniella* previously deposited at the herbaria FMB and K (Thiers, 2021) were studied, including digitised type and reference specimens available at JSTOR Global Plants (http://plants.jstor.org/), Reflora (http://reflora.jbrj.gov.br/reflora/herbarioVirtual), MO (https://www.tropicos.org/), NYBG (http://sweetgum.nybg.org/science/vh/), and biovirtual (http://www.biovirtual.unal.edu.co/es/colecciones/search/plants/). These specimens included typological material of all accepted species in *Magoniella* (**Supplementary Table S3**).

We investigated six characters of ripe fruits, including ripe fruit length, perianth tube length, perianth tube width, fruit sepal length, fruit sepal width, and bracteole length. These measurements were obtained from digital images using the ImageJ v. 2.8.0 software (Schneider et al., 2012). A total of 19 *Magoniella* specimens were measured, with three different fruits studied for each specimen (*n* = 74 fruits). To assess biologically significant differences, we employed the approximate Two-Sample Fisher-Pitman Permutation Test (Fisher, 1936). Given that our comparative DNA and morphometric analyses clearly indicated that the studied specimens diverge from individuals of closely related species (see Results below), we followed Pendry (2004) terminology to construct a morphological description of the new species. The putative new species was diagnosed based on a unique combination of identified features (Donoghue, 1985). We derived the geographical distribution ranges of *Magoniella* from the examined specimens in this study and botanical monographs (**Supplementary Table S4**, Pendry, 2004).

### Preliminary conservation status

We estimated the conservation category of the new taxon by calculating its extent of occurrence (EOO) and area of occupancy using the GeoCAT tool (Bachman et al., 2011) and following the IUCN Red List Categories criteria (IUCN Standards and Petitions Subcommittee, 2013).

## Results

### Phylogenetic relationships in Triplaridae

**Figure 1:**
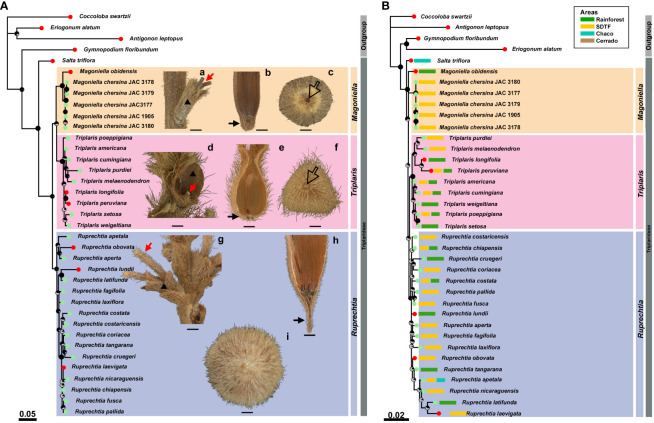
Maximum likelihood phylograms inferred from combined **(A)** ITS + *lfy*2*i* nuclear loci and **(B)** combined *matK* + *ndhF* + *rps*16-*trnK* + *ndhC*-*trnV* plastid loci. Fully black pie charts at nodes indicate nodes attaining maximum statistical support. Circles preceding species names indicate potential outlier (red circle) and congruent (green circle) terminals. Pictures in front of each clade show morphological characters of the partial infructescence and ripe fruits of the new species plus representative species of *Ruprechtia* and *Triplaris*. *Magoniella chersina* (FMB-124471): (a) Partial infructescence with pedicels (red arrow) longer than tubular bracteole (black triangle). (b) Longitudinal view of the perianth tube of ripe fruits with the base abruptly taper to scars, showing a narrow, somewhat sharpen base of up to 2.5 mm long (black arrow). (c) Inferior view of the basal part of the fruiting perianth tube (open arrow indicates scar from abscised pedicel). *Triplaris weigeltiana* (FMB-24212): (d) partial infructescence with bracteole fissured abaxially (black triangle) and a single pedicel (red arrow). (e) Longitudinal view of ripe fruits showing the base of the perianth tube truncate (black arrow). (f) Inferior view of the basal part of the fruiting perianth tube (open arrow indicates scar from abscised pedicel). *Ruprechtia ramiflora* (FMB-124421): (g) Partial infructescence with two pedicels (red arrow) subtended by a single tubular bracteole (black triangle). (h) Longitudinal view of ripe fruits showing a perianth tube base extending into a stalk (black arrow). (i) Inferior view of the basal part of the fruiting perianth tube (open arrow indicates scar from abscised pedicel). Black bars represent 1 mm. Geographical distribution in Neotropical biomes of sampled species is shown in front of tree terminals with coloured boxes preceding species names (see **Supplementary Figure S2**).

The combined nuclear alignment contained 2,076 base pairs, with 1,204 variable and 451 parsimony informative sites. The combined plastid alignment contained 3,363 base pairs, of which 171 were parsimony informative. ML analyses independently inferred from the concatenated nuclear (**Figure 1**a) and plastid (**Figure 1**b) datasets recovered the monophyly of Triplarideae [maximum likelihood bootstrap support (MLBS): 100%–99%, respectively]. Within the Triplarideae, three strongly supported clades were recovered, supporting the monophyly of *Triplaris* and *Ruprechtia s.s*. (**Figure 1**).

A visual comparison between the nuclear and plastid phylogenies revealed multiple topological incongruences. To quantify the level of topological discordance, we conducted an analysis in PACo, which revealed that 15 terminals were potentially conflicting. Twelve of these terminals belonged to *Ruprechtia* s.s. and three to *Triplaris* (two outliers were misclassified by PACo as conflicting: *Ruprechtia pallida* and *Ruprechtia fusca*). In particular, the nuclear phylogeny placed *Salta triflora* as sister to the remainder of the tribe, where *Magoniella* and *Triplaris* clustered in a poorly supported clade (MLBS: 56%) sister to *Ruprechtia* (**Figure 1**a). In contrast, the plastid phylogeny recovered *Magoniella* as a polyphyletic group, placing *M. obidensis* as sister to *Ruprechtia*, *Magoniella* and *Triplaris*, and with the relationship of *Triplaris* + *Ruprechtia* recovered as poorly supported (MLBS: 42%; **Figure 1**b). Because the incongruent terminals were exclusively placed in poorly supported branches (**Figure 1**), no data were excluded from the nuclear–plastid supermatrix used for ML phylogenetic inference.

**Figure 2:**
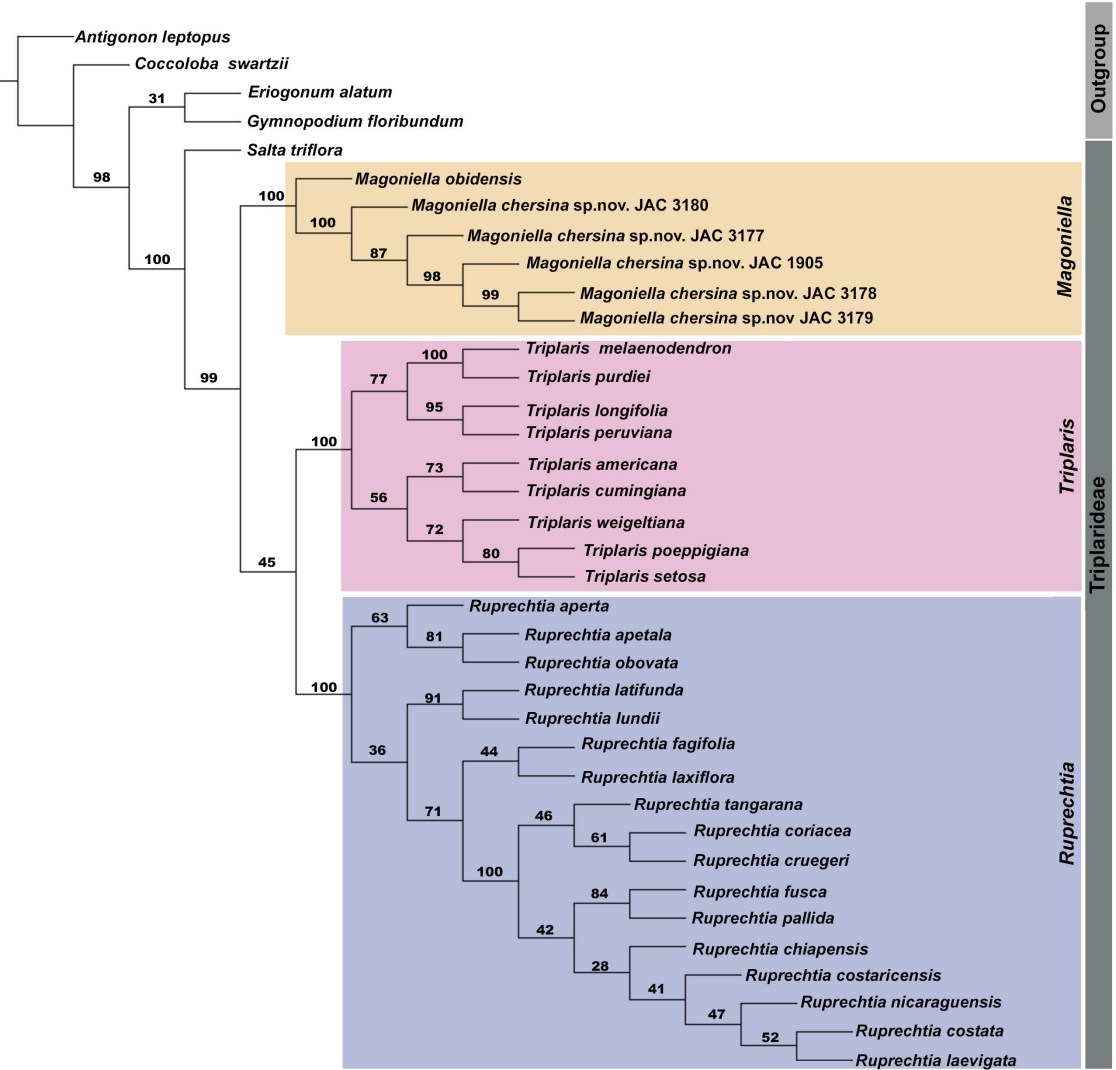
Maximum likelihood tree showing the phylogenetic relationships of Triplaridae inferred from a nuclear-plastid supermatrix (ITS + *lfy*2*i* + *matK* + *ndhF* + *rps*16*-trnK* + *ndhC-trnV*). The values above branches indicate bootstrap support percentages.

The ML analysis from the nuclear–plastid matrix yielded an overall strongly supported phylogeny (**Figure 2**). The results presented below are therefore based on this phylogeny. Here, the tribe Triplarideae was recovered as monophyletic (MLBS: 100) and segregated into three main lineages corresponding to *Magoniella*, *Triplaris* and *Ruprechtia*. *Salta triflora* was placed as sister to these three maximally supported clades. Within Triplaridaea, *Magoniella* clustered in a maximally supported clade (MLBS: 100); *Ruprechtia s.s.* was recovered as sister to *Triplaris* in a poorly supported clade (MLBS: 45; **Figure 2**).

### Geographical distribution and morphological differentiation

**Figure 3:**
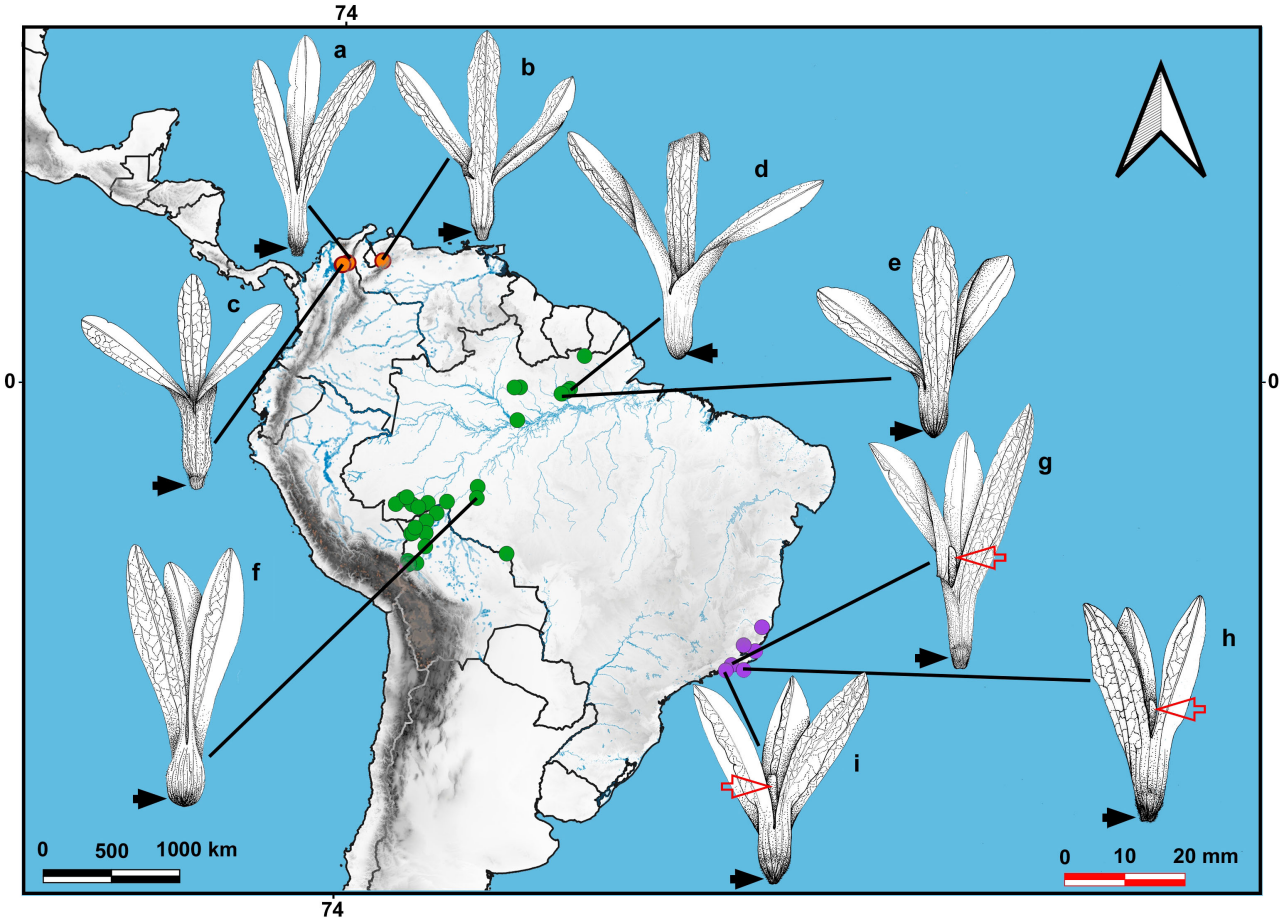
Geographical distribution and ripe fruit shape variation of *Magoniella* species. Fruit illustrations are based on herbarium specimens, as follows: *Magoniella chersina*: (a) Allen 929 (K-H3651/6019). (b) Aguilar-Cano 1905 (FMB-124471). (c) Pittier 13302 (MO-983676). *Magoniella obidensis*: (d) Ducke 2899 (F-602321). (e) Ducke 19542 (K). (f) Prance 6991 (K). *Magoniella laurifolia*: (g) Petroa 6703 (K). (h) Sucre 3512 (K). (j) Maquete 634 (K). Illustrations drawn by M. Paula Mendivil to a uniform scale (red–white bar). Key features, including the base of the perianth tube (black arrow) in the fruit abruptly or gradually tapering to the abscission zone, resulting in a narrow, somewhat sharp or broad obtuse base, and the sepaloid fruiting petals (red arrow), distinguishes *M. chersina* (orange dots) from *M. laurifolia* (purple dots) and *M. obidensis* (green dots).

**Figure 4:**
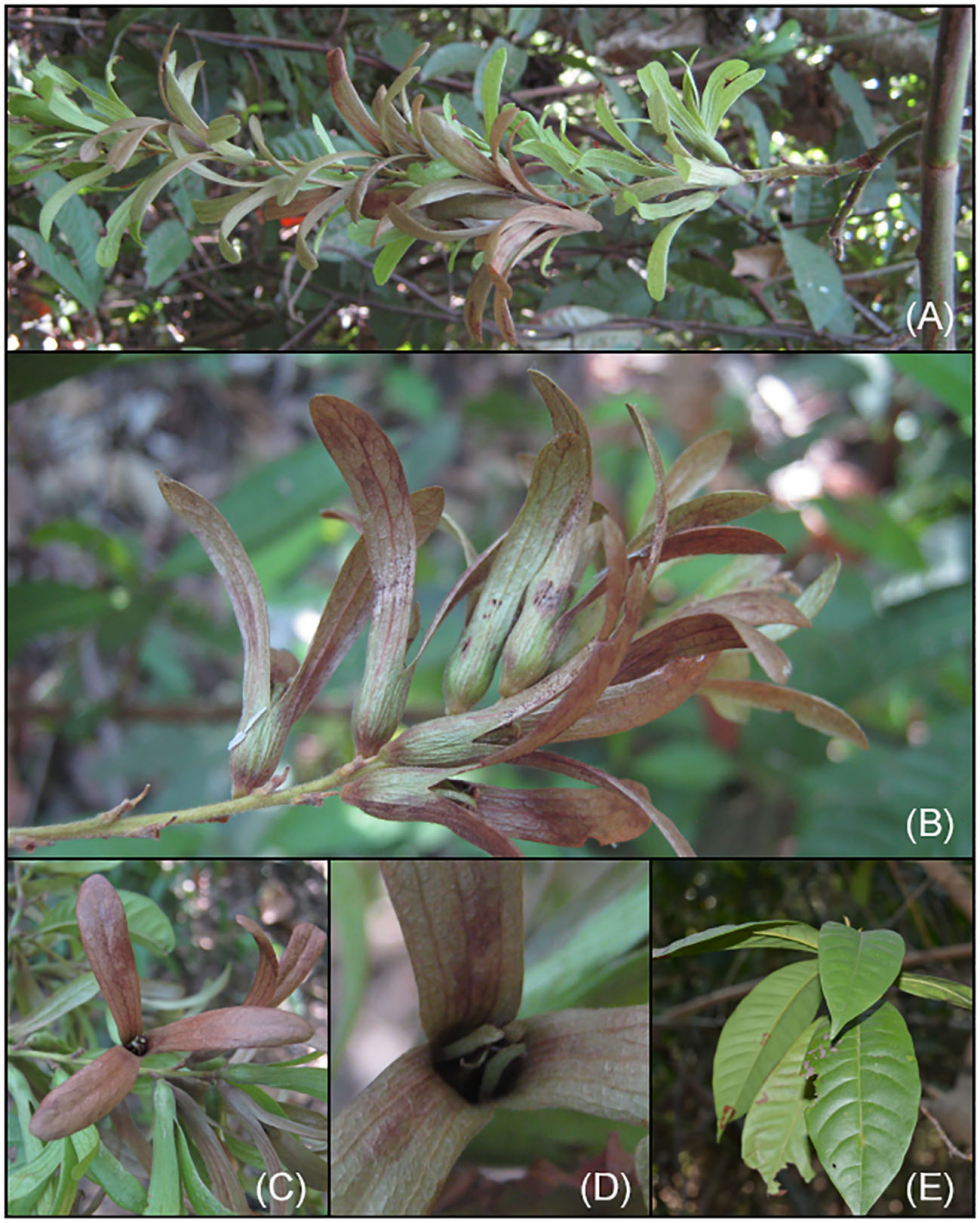
*Magoniella chersina* Aguilar-Cano & O.Pérez. **(A)** Habit and fruiting branch. **(B)** Infructescence. **(C)** Fruits seen from above showing sepals. **(D)** Fruit detail seen from above showing petals. **(E)** Leaves on the abaxial side of a juvenile individual.

We found distinct geographical ranges among the three *Magoniella* species. Our geographical distribution records confirmed that *M. obidensis* and *M. lauriflora* are exclusively found in the tropical rainforests of the Brazilian and Bolivian Amazon basin, and the Atlantic Forest in southeastern Brazil, respectively. In contrast, the specimens of the newly described species, *M. chersina*, are limited to the STDF of the Colombian and Venezuelan Caribbean (**Figures 1**b, **3**, **4**). This new species stands out for its widely disjunct northern distribution, which is notable both for the significant geographical distance from the other two species and its presence in an eco-physiologically distinct biome.

**Figure 5:**
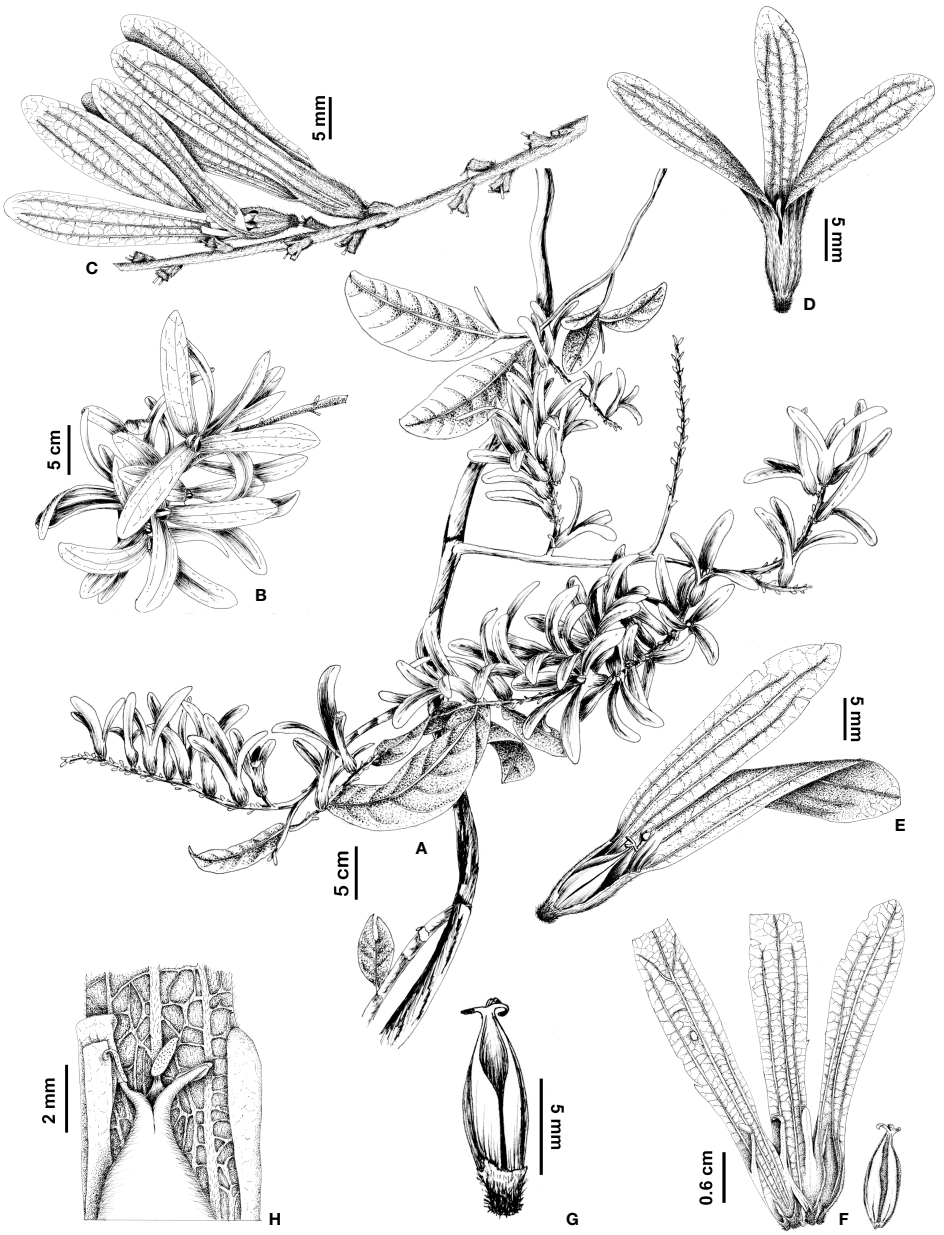
*Magoniella chersina* Aguilar-Cano & O.Pérez. **(A)** Habit and fruiting branch. **(B)** Infructescence. **(C)** Partial infructescence. **(D)** Fruit. **(E)** Longitudinal section of fruit. **(F)** Fruit with the sepals opened and seed. **(G)** Seed with basal extension of the calyx remnant. **(H)** Zoom-in on a seeds showing the upper portion. Illustration by Camila Pizano from the holotype specimen.

**Figure 6:**
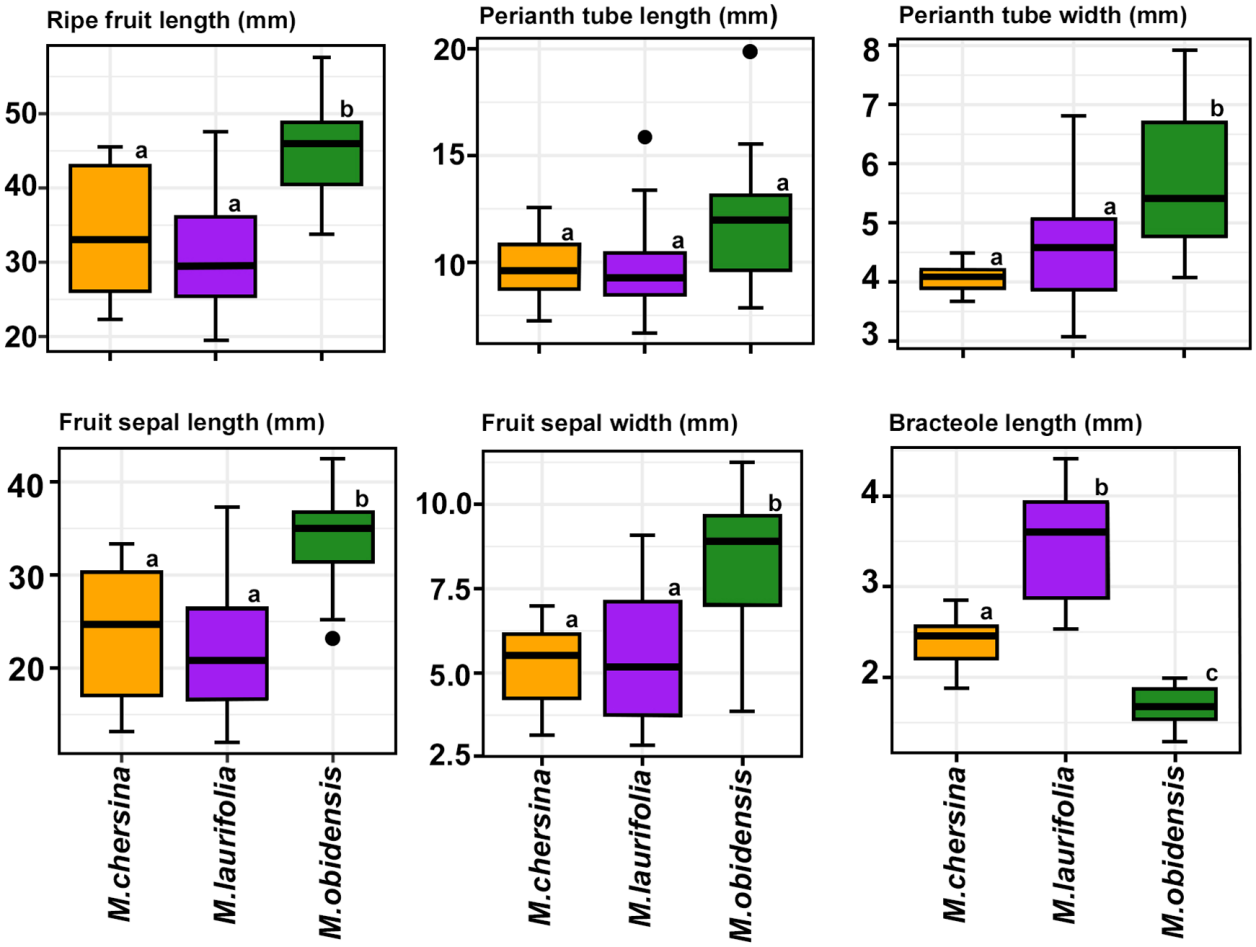
Differences in six morphological characters of ripe fruits among species of *Magoniella* (*M. chersina*, *M. laurifolia*, *M. obidensis*). The lowercase letters (a-c) above the boxes indicate statistically significant differences (there were no significant differences between species that are marked with the same letter). Note that bracteole length was the discriminatory trait for the three species.

The new species shares several characteristics with the other two species (*Magoniella obidensis* and *M. laurifolia*), including a strict lianaceous habit, leaf shape, hollow stems, and large green fruits with chartaceous reddish sepals (**Figures 4A–D**) (Pendry, 2004; Sanchez & Kron, 2011). However, it possesses a unique combination of fruit traits not found in any *Magoniella* species (**Figures 3a–c**, **4**, **5**; see species diagnosis and key to the species in the *Taxonomic treatment* section). In our comparative analysis, we conducted the Fisher-Pit permutation test implemented on measurements of six morphological characters for all *Magoniella* species. The results of the our comparative analysis revealed that bracteole length differed significantly between *M. chersina* and *M. laurifolia*, *M. chersina and M. obidensis, and M. laurifolia* and *M. obidensis* (**Figure 6**, **Supplementary Table S5**; *p* ≤ 0.05) and that *M. obidensis* has significantly larger fruits than those found in the new species and in *M. laurifolia* (**Figure 6**, **Supplementary Table S5**; *p* ≤ 0.05). The remaining five characters of *M. chersina* were significantly different from *M. obidensis* (**Figures 6**, **Supplementary Table S5**; *p* ≤ 0.05), but not from *M. laurifolia* (**Figure 6**, **Supplementary Table S5**; *p* ≤ 0.05). In summary, based on the morphological differences, disjunct geographical range, and phylogenetic divergence of the individuals found in the SDTFs of Colombia and Venezuela compared to their sister species, we formally describe these populations as a new species to science (see *Taxonomic treatment*).

## Discussion

Our phylogenetic analyses revealed that *Magoniella chersina* and *M. obidensis* form a strongly supported clade, corroborating their exclusion from *Ruprechtia*, as previously reported by Sanchez and Kron (2011). Despite having a limited sample of *M. obidensis* in our analyses, these results further establish the placement of the new species within the monophyletic genus *Magoniella* (**Figure 2**). Moreover, while we were unable to include sequences from *M. laurifolia* in our phylogenetic analysis due to their absence in the NCBI database and our inability to collect specimens in the field or sequence DNA from historical specimens, the new species demonstrates a clear relationship with *M. laurifolia.* This is supported by shared morphological features (dense indumentum on the principal axis of the inflorescences, bracts, and base of the perianth tube). However, *M. chersina* can be distinguished by a set of fruit morphological characters, whereby its pedicels are longer than the bracteoles, clearly visible after the ripe fruits have fallen (**Figure 5C**) (vs. shorter than the bracteoles, not noticeable after the ripe fruits have fallen in *M. laurifolia*) and fruits with shorter linear petals, 10–11 mm (**Figures 5D, F**) (vs. petals sepaloid, variable, 17–30 mm in *M. laurifolia*).

The presence of the strict liana habit, not observed in any other species of Triplarideae, stands as a synapomorphy of *Magoniella* as previously proposed by Sanchez and Kron (2011) based on the species known at that time. The five accessions of *M. chersina* coalesced into a highly supported clade within *Magoniella* (**Figure 2**). Similar clustering patterns have been recovered in various unrelated lineages characterised by high levels of endemism across distinct regions of Neotropical SDTFs (Pennington et al., 2004). Our study contributed further evidence of the distinct phylogeographic structure often associated with STDF lineages, characterised by having long internal branches and terminal with short branch lengths (Pennington et al., 2004). These findings reinforce the notion that STDF species exhibit marked isolation and dispersal limitations, leading to *in-situ* speciation and strong niche phylogenetic conservatism (Pennington et al., 2004; Pennington and Lavin, 2016; Särkinen et al., 2011b).

The discordance in nuclear-plastid tree topologies observed in our datasets aligns with findings in numerous angiosperm lineages (Pérez-Escobar et al., 2017, 2021; Stull et al., 2023; Zuntini et al., 2024) (**Figure 1**). Here, the nuclear phylogeny strongly reflects relationship patterns supported by morphological evidence, such as partial infructescence, fruiting perianth tube and basal part of the fruiting perianth across the three genera. The *Magoniella* and *Triplaris* clades includes species with the base of perianth tube not extending into a narrow stalk terminated by scar from the abscised pedicel (**Figures 1A**, b, e). In contrast, the *Ruprechtia s.s* clade includes species with the base of the perianth tube extended into a pedicel-like structure, a stalk (**Figures 1**a–h). Notably, the *Magoniella* clade differs from other two major Triplarideae clades in having the basal part of the fruiting perianth tube either abruptly or gradually tapering to the abscission zone, resulting in a narrow or broad obtuse base (**Figures 3a-i**). This base never extends into a stalk, as in the *Ruprechtia* clade, or terminates abruptly into a truncate base, as in the *Triplaris* clade (**Figures 3a-i**).

Interestingly, the plastid tree appears to match well with the geographical distributions of the two lineages within *Magoniella*. *M. obidensis* occurs in humid forest of the Amazon basin, whilst *M. chersina* is exclusively distributed in disjunct locations within the SDTFs of the Colombian and Venezuelan Caribbean (**Figure 1B**). Plastid trees reflecting geographical clustering patterns at shallow phylogenetic levels as compared to nuclear trees is a phenomenon that has been reported for several plant lineages (e.g., Araceae: Nauheimer et al., 2012; Asteraceae: Fehrer et al., 2007; Orchidaceae: Pérez-Escobar et al., 2017; Polemoniaceae: Rose et al., 2020), including legumes (Gu et al., 2024). Here, the often-localised female gamete dispersal mode in angiosperms compared with the longer travelling male gametes could explain why uniparentally inherited organellar genomes better track geographical distribution patterns than phylogenies produced from biparentally inherited genomes (i.e., nuclear). Although the nuclear results show that *M. chersina* is distinct from sister *M. obidensis* (**Figure 1B**), when considered alone, the plastid phylogenomic evidence further strongly supports the hypothesis that *M. chersina* is indeed a divergent lineage from *M. obidensis. Magoniella chersina* shows no geographical overlap with the other two species in the genus (**Figure 1B**).

Colombian seasonally dry forests are estimated to have originated around 20,000 years ago, likely during the Last Glacial Maximum (Werneck et al., 2010), and have served as a cradle of rapidly speciating orchid lineages (Pérez-Escobar et al., 2024). However, this chronology contradicts phylogenetic evidence for dry-adapted Andean legumes, which suggests that legume lineages have been geographically isolated for several million years, spanning from 4.0 to 18.8 million years ago (da Cruz et al., 2018; Särkinen et al., 2011a). Furthermore, Pennington et al. (2004) suggested that *Ruprechtia* species from the Caribbean coast of Colombia and Venezuela comprise a lineage of post-Pleistocene diversification. This discrepancy merits further investigation, including a molecular dating analysis of tribe Triplaridae. This analysis will enable an evaluation of whether *Magoniella chersina* could have evolved in pockets of dry vegetation older than the Last Glacial Maximum, or initially thrived in humid forests and gradually adapted to the dry conditions of Colombian seasonally dry forests. As Antonelli et al. (2018) have demonstrated, transitions from forest to open habitats have been commonplace throughout the history of Neotropical plant evolution, which could provide an explanation for the unique case of *Magoniella chersina*.

### Taxonomic treatment

#### 1. *Magoniella chersina* Aguilar-Cano & O.Pérez **sp. nov.**

##### Type

COLOMBIA, Cesar, municipio de Chimichagua, vereda Santo Domingo Hacienda Rincón Grande. Fragmento de bosque inundable en las márgenes de la Ciénaga de Zapatosa, vegetación secundaria semiabierta dominada por *Attalea butyracea* y *Cecropia peltata*, 9°14’5.74”N 73°50’10.14”W, 29 m, 3 March 2015, José Aguilar Cano, Sandra Medina & Luis Morelo 1905 (holotype FMB-124471!, isotypes COL-615779, K) (**Figures 4**, **5**).

##### Diagnosis

The new species differs from the other *Magoniella* species by the basal part of the fruiting perianth tube abruptly tapering to abscission zone, resulting in a narrow, somewhat sharp base of up to 2.5 mm long, completely covered with densely sericeous-pubescence of erect, longer trichomes and pedicels longer than the tubular bracteole, clearly visible after the ripe fruits have fallen. The nuclear ribosomal ITS region differs from that of *M. obidensis* by a) the **GT → TA** (176–178 bp), **TC → CT** (217–219 bp), **TA → AT** (274–276 bp), **CA → AC** (277–279 bp) substitutions; b) 5-point transitions (**T → C**: 445 bp, 492 bp, 592 bp; **C → T**: 225 bp,; **A → G**: 574 bp); and c) 1 point transversion (**G → T:** 670 bp). Additionally, the *lfy*2 gene of *M. chersina* differs from that of *M. obidensis* by containing 5-point transitions (**A → G:** 250 bp; **G → A:** 350 bp; **C → T:** 488 bp, 954 bp; **T → C:** 1,173 bp) and 4 transversion (**G → T:** 426 bp, 584 bp; **T → G:** 820 bp; **A → T:** 926 bp).

##### Description

Liana, young individuals erect to 50 cm tall; tap root; twigs terete, hollow, green when very young and pale griseous with age and inhabited by ants in dilated nodes, slightly lenticellate, young branches slightly pubescent, trichomes appressed, golden-brown, glabrescent to completely glabrous with age.

**Stipules** persistent ochreas, entire, 5–6 (–7) mm long, encircling the twigs, caducous with age, slightly pubescent especially towards the margin, long trichomes appressed, glabrescent with age. **Leaves** alternate, membranaceous to sub-chartaceous, elliptic, leaf blade 18–27.5 × 7–10.5 cm in erect young individuals and 6.5–18.5 × 2.2–7 cm in lianescentes adult individuals, base cuneate, sometimes short decurrent at petiole, apex acuminate, margins moderately undulate, adaxial leaf surface glabrous, abaxially glabrescent, sometimes with minute dark glands, pubescent especially along the midrib and secondary veins, short trichomes appressed; venation brochidodromus, midrib elevated adaxially, secondary veins prominent abaxially, 12–23, veins per side, slightly sunken adaxially with the lamina bullate, tertiary venation faint and dense scalariform-reticulate; petioles 0.5–1.5 cm long, glabrous adaxially, pubescent abaxially, trichomes appressed. **Male inflorescences** axillary, usually simple, rarely branched at the base, 13–26 cm long; flowers arranged in partial inflorescences along the principal axis, internodes to 3–5 mm long, densely pubescent, erect or wavy short trichomes; each partial inflorescences 3–5 flowered, bearing by a minute bract, 1–1.5 mm long, acute, densely pubescent, appressed trichomes. **Male flowers** notably pedicellate, 2.7–3.0 mm long, *remaining* after the flowers have been fallen, glabrescent; subtended by tubular bracteoles, 2 mm long, glabrescent; sepals 3, ovate, up 1.0 mm long, pubescent; petals 3, ovate, 0.8–1.3 mm long, sparsely pubescent; stamens 9 in two whorls (6 outer and 3 inner), filaments 0.5–0.9 mm long; anthers 0.35–0.40 mm long. **Female flowers** not seen. **Infructescence** axillary simple, when terminal branched, up to 20 cm long; lax, fruits arranged in partial infructescence along the principal axis, internodes 2–10 mm long, densely pubescent, erect or wavy short trichomes; partial infructescence bearing by a bract, to 2 mm long, acute, densely pubescent, appressed trichomes, each partial infructescence with 2 fruits, subtended by single tubular bracteole, 1.9–2.8 mm long, densely pubescent, appressed trichomes; pedicels longer than the bracteoles, 2.4–2.9 mm long, densely sericeous at the base, glabrous towards the apex, remaining after fruits have been fallen. **Fruits** winged achenes, 37–41 mm long, completely green to green with variegate reddish immature, completely red when ripe; perianth tube of ripe fruits narrowly elongate, campanulate, 6–8 mm long, sparsely pubescent, appressed trichomes, the basal part of the fruiting perianth tube shortly tapering to abscission zone, resulting in a narrow, somewhat sharp base up to 2.5 mm long, completely covered with densely sericeous-pubescence with erect, longer trichomes; sepals of ripe fruits diverging from a slightly constricted zone above of seed, 25–35 × 6–9.5 mm long, obovate, chartaceous, abaxially glabrescent, adaxially glabrous, main and secondary venation evident, tertiary venation, densely reticulate; fruiting petals persist, linear, 10–11 mm long, oblong, adnate to the inside of the perianth tube, the free part 5.5–6.5 × 0.7–0.8 mm long, abaxially glabrescent, adaxially glabrous; staminodes ca. 0.5 mm long in fruit; disk glabrous; seeds three-lobed, 11–12 mm long, smooth, glabrous, stigmas persist in fruit, linear to ellipsoid 0.8–1.0 mm long.

##### Additional specimens examined (paratypes)

COLOMBIA, Cesar, municipio de Chimichagua, vereda Santo Domingo, Vereda Santo Domingo. Hacienda Rincón Grande. Fragmento de bosque inundable en las márgenes de la Ciénaga de Zapatosa, vegetación secundaria semiabierta dominada por *Attalea butyracea* y *Cecropia peltata*.9°14’5.74”N 73°50’10.14”W, 29 m, 15 de abril 2016, José Aguilar-Cano, Sandra Medina, Felipe Villegas & Juan Carlos Roble 3177 (FMB-124472; COL); 9°14’5.74”N 73°50’10.14”W, 29 m, 15 de abril 2016, José Aguilar-Cano, Sandra Medina, Felipe Villegas & Juan Carlos Roble 3180 (FMB-124474); vereda Ojo de agua. Predios de Asopeagro, vegetación secundaria abierta asociada a las márgenes de la Ciénaga de Zapatosa, compuestas por densos entramados de bejucos y arbustos, el único elemento emergente corresponde a la palma *Attalea butyracea*”. 9°15’32.98”N 73°46’38.39”W, 27 m, 15 abril 2016, José Aguilar Cano, Sandra Medina, Felipe Villegas & Juan Carlos Roble 3178 (FMB-124473); 9°15’32.98”N 73°46’38.39”W, 27 m, 15 abril 2016, José Aguilar-Cano, Sandra Medina & Felipe Villegas 3179 (FMB-124475); municipio de Chiriguaná, Poponte, [9°23’38.90”N 73°22’49.32”W, 93 m], 1 de febrero 1925, C. Allen 929 (K). VENEZUELA, Trujillo, between Quebrada Seca bridge and Motatán, [9°32’22.56”N 70°36’37.76”W, 400 m], 1 de febrero 1929, R., Pittier 13302; 13299 (F, GH, M, MO, NY, VEN).

##### Distribution and ecology

*Magoniella chersina* is restricted to the Neotropical dry forest (SDTF) nuclei of the Caribbean coast of Colombia and Venezuela. In Colombia, it is known from three localities in Cesar; two populations *ca* 7 km apart (including the type locality) exist in Chimichagua municipality, situated at the north of the Zapatosa floodplain lake complex. These regions are 15–29 m in elevation, and represent one of the largest freshwater marshes in Colombia. Notably, this area has been designated as a Ramsar wetland of conservation priority. An additional population is located in the Chiriguana municipality, at the lower part of the western foothills from the Perijá massif, northernmost of the Cordillera Oriental, at *ca* 46 m of elevation.

**Figure 7:**
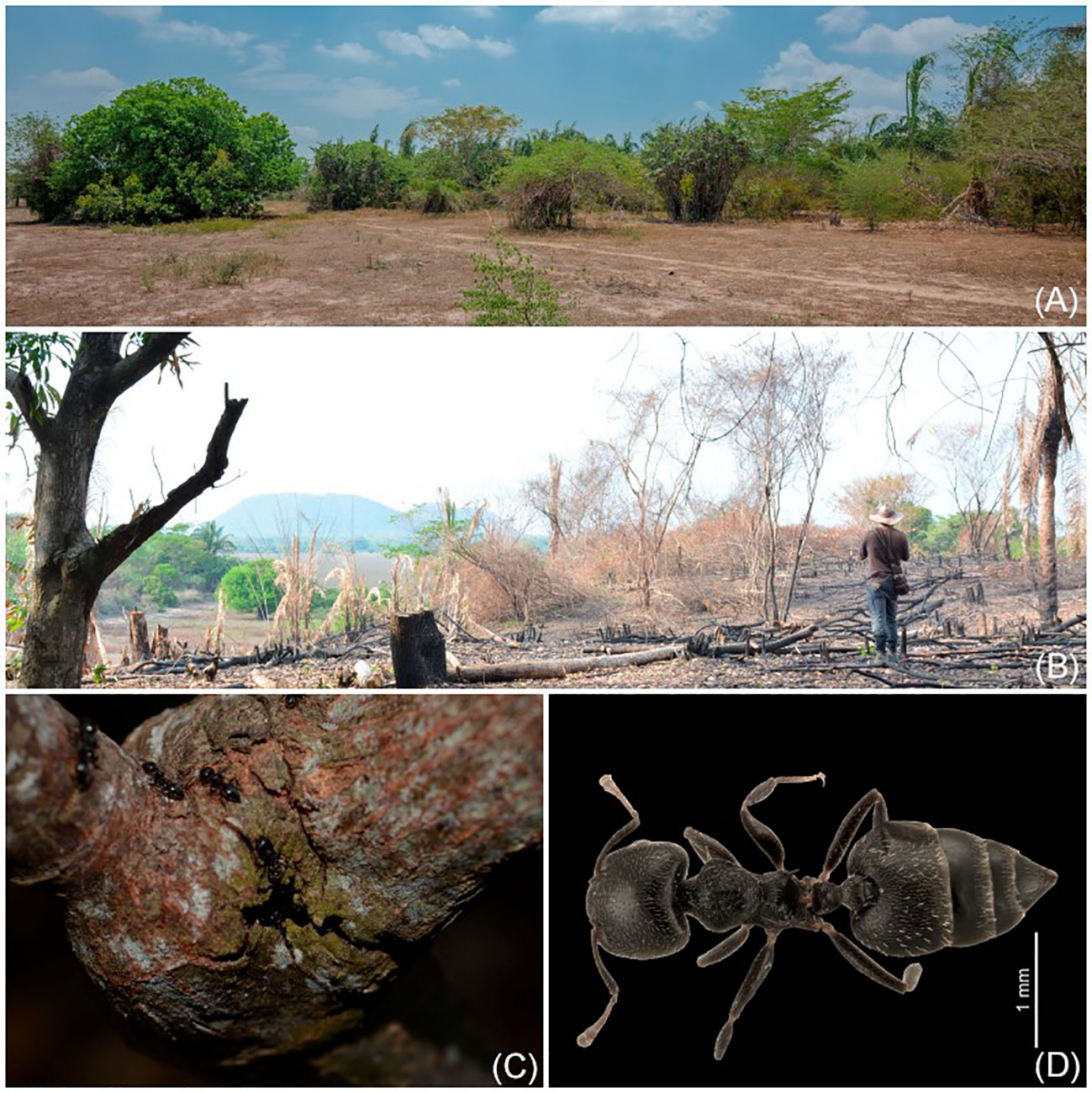
**(A)** Habitat of *Magoniella chersina* in its the type locality. **(B)** Habitat of *M. chersina* at the north of the Zapatosa floodplain lake complex in Cesar (Colombia), affected by slash-and-burn agriculture. **(C)** Stems with hollow nodes inhabited by ants. **(D)** *Crematogaster erecta* Mayr, 1866, [IAvH-E-261079; IAvH-E-261080], dorsal. Photos: Felipe Villegas **(A, B)**, José Aguilar-Cano **(C)**, Jhon Neita **(D)**.

The populations from the holotype and paratype were collected in seasonally inundated flooded areas near the wetland edges in quite disturbed forest openings, with sites mostly dominated by the palm *Attalea butyracea* and the pioneer tree *Cecropia peltata*, on which the new species climbs (**Figures 7A, B**). Burning for agriculture seemed to be frequent in the type locality (**Figure 7B**), potentially posing a threat to the new species and its native ecosystem. The record of the Perijá massif was collected almost a century ago by C. Allen in 1925 and deposited in the K herbarium. Several researchers observed the material of the previously undescribed species but did not formally describe it, incorrectly identifying it as *Ruprechtia laurifolia*, *R. obidensis*, and *Triplaris scandens*. Similarly, Henri Pittier collected two specimens in 1929, which were initially identified as *M. obidensis*is, and these were found in the lower part of the western foothills of the northern areas in the Venezuelan Cordillera of Mérida, near to the Maracaibo Lake (Pendry, 2004). As a result, all known populations of the new species are disjunct in their distribution compared to populations of *M. obidensis* and *M. laurifolia*, which are primarily restricted to the Amazon basin and the Atlantic Forest, respectively (**Figure 3**). The geographical range of *Magoniella chersina* falls completely within the distinctive dry forest vegetation (**Figure 1**b), and does not share its habitat with any close relatives in the Caribbean coast of Colombia and Venezuela (**Figure 3**). Moreover, none of the species of *Ruprechtia s.s.* and *Triplaris* that occur in the neotropical SDTF nuclei could be confused with *M. chersina*. Therefore, the new species is considered endemic to the Caribbean coast of Colombia and Venezuela, representing the first record of the genus *Magoniella* in the flora of those two countries.

Hollow internodes have been found in seven species of *Ruprechtia*, but only *R. tangarana*, a western Amazonian species, has been reported to be inhabited by *Pseudomyrmex* ants (Brandbyge and Øllgaard, 1984; Brandbyge, 1989). Additionally, *R. cruegeri*, *R. laurifolia*, *R. lundii*, *R. maracensis*, and *R. latifunda* appear to host ants, but the species found belong to the genus *Crematogaster* (Formicidae, Myrmicinae) (Pendry, 2004; Sanchez and Kron, 2011). In *Magoniella*, only *M. obidensis* has been reported to harbor ants (Pendry, 2004; Sanchez and Kron, 2011). During our study, we observed the presence of *Crematogaster distans* (Mayr 1870) (**Figure 7D**) associated with ten vines of the new species. These ants were found in hollow internodes and in the unvascularised areas near the nodes, where the stem widens and develops cracks, allowing ants to enter and exit the host plant (**Figure 7C**).

While opportunistic ants might be found in any plant with hollow cavities (Mayer et al., 2014), in the case of *Triplaris*, a close relative of *Magoniella*, there appears to be a tendency for related species of *Pseudomyrmex* ants to prefer the same plant species (Ward, 1999; Torres and Sanchez, 2017). Most ants show a broader host range, indicating that there are also varying degrees of ant-host specificity (Sanchez, 2015). Although it is uncertain whether these ants offer any protection from herbivory in *Magoniella chersina* or are obligate mutualists, we found them always nesting inside the plants. Therefore, the association between *Magoniella chersina* and *Crematogaster distans* cannot be definitively classified as obligate or specific mutualism, and further collections are warranted to clarify the nature of the interaction and its mode of evolution between these two organisms.

##### Phenology

The fruits of the new species were collected in March and historical specimens were collected in fruit in February. Flowering does not seem to be annual, given that in 2015 the plant was fruiting, but the following year (April 2016) it was found in a sterile state and with abundant regeneration from the previous year’s flowering and fruiting. In contrast, *Magoniella obidensis* and *M. laurifolia* typically undergo flowering and fruiting in June and October. These differences in phenological seasonality between *M. chersina* and its sister species are noteworthy.

##### Dispersal

The winged achenes of the Triplarideae species develop from the perianth, and their wind dispersal is associated with different levels of flight performance, dependent on the relationship between symmetry, wing angle, and fruit weight. Generally, mature fruits are dispersed a few meters from the mother plant (Pendry, 2004; Finalé, 2016). The winged achenes in *Magoniella* (**Figure 3**) also appear adapted to wind-dispersal, yet their dry wings and low density also allow them to float on the water surface and to be transported by water currents. Therefore, we propose that the fruits of *M. chersina* (**Figures 3**, **5**) are mainly dispersed by water, the primary dispersal method for species in riverine forests that lack or have limited long-distance wind dispersal. While most dry forest lianaceous species rely on wind for dispersal (Gentry, 1995), water dispersal is more commonly associated with rheophytic plants in swamps, watercourses, and shorelines, playing a limited role in the dispersal of seeds and fruits of terrestrial plants (van der Pijl, 1982; Weberling, 1989). In its wetland edge habitat, the new species likely relies on water for its dispersal, with wind potentially important only for short-distance dispersal.

##### Etymology

The epithet is derived from the Greek root *chersin* (=dry). This choice reflects current knowledge of the species’ habitat preference for dry areas, unlike the other two species of the genus that inhabit humid rainforests.

##### Conservation status

This species is endemic to the STDF of the Colombian and Venezuelan Caribbean. We observed that two of the four currently known populations of the new species occur in the Zapatosa floodplain lake complex, a Ramsar wetland conservation area corresponding to the type localities. These two populations have a combined total of *ca* 15 adult individuals, with a continuous recruitment of new individuals from seed. Young individuals are densely aggregated in populations of between 25 and 75 young individuals, clustered around parent plants, showing that populations of the new species seem to have high seedling recruitment. However, seasonally inundated flooded areas near the wetland edges are highly fragmented and the few remnants that persist are surrounded by roads, croplands, and cattle pastures (Rivera et al., 2013) and exposed to continuous threats, such as habitat loss because of non-sustainable activities, logging, fire, and illegal settlements (**Figures 7A, B**).

Two additional herbarium specimens were collected in the 1920s in areas that now appear on satellite imagery to have little forest cover left, with land use changes, including agriculture, mining, or urban settlements. New surveys at the known localities for this species are recommended to confirm the species’ distribution and establish the current population size and trends.

Almost two-thirds of dry forest in Colombia and Venezuela have been lost, primarily due to agriculture and cattle grazing, and less than 5% is under protection (Fajardo et al., 2005; Rodríguez et al., 2008; García et al., 2014; González-M et al., 2018). This means that modern threats in this area are common and frequent, and the habitat of this species is in continuous decline. The EOO of this species has been estimated at 2,265 km^2^, below the threshold for the Endangered category, and its habitat has been continually declining, with less than five current locations. However, information on populations is incomplete, and the species could not be assessed based on its population size and decline. We suspect that the population has been declining, based on the species’ habitat preference and habitat loss in its region of occurrence. We suggest that *M. chersina* should be considered as Endangered [**EN B1ab (iii**)], due to its restricted range and considering the lowest number of locations combined with an inferred continuing decline in its habitat. We recommend that a formal IUCN Red List assessment is carried out swiftly.

#### 2. *Magoniella obidensis* (Huber) Adr. Sanchez

**Basionym:** *Ruprechtia obidensis* Huber in Bol. Mus. Paraense Nat. Hist. 5: 344. 1909. TYPE: BRAZIL. Para: Obidos, capueira, 31 Jul 1902, A. Ducke 2899 (lectotype, here designated: MG 002899!; isolectotypes: BM 571400!, F 602321 (photo & fragment)!, G 00437689!. *Ruprechtia apetala* var. *sprucei* Meisn. in C.F.P.von Martius & auct. suc. (eds.), Fl. Bras. 5(1): 57. 1855. —TYPE: Brazil. Para: circa Santarém, Jun 1850, Spruce s.n. (holotype: G 00437688!, Field Museum negative no. 7414: F, GH 00036827! GOET 006066! NY 00260344!. *Ruprechtia macrocalyx* Huber, Bol. Mus. Goeldi Hist. Nat. Ethnogr. 5: 345. 1909. —TYPE: Brazil. Para: Faro, capoeira, 27 Aug 1907, Ducke 8540 (lectotype, here designated: MG 008539!; isolectotypes: BM 000571404!, F 651468 (photo & fragment)!, G 00437692!. *Ruprechtia scandens* Rusby, Mem. New York Bot. Gard. 7: 237. 1927. —TYPE: Bolivia. El Beni: Huachi, head of the Beni River, 1800 ft, 18 Aug 1921, Rusby & White 972 (lectotype designated by Pendry, 2004: NY 00260320! (two sheets); isolectotypes: B 100250422, BKL 00004118, GH 00036834!, GH 00036833, MICH 1111770!, NY 00260321!, NY 00260319!, K 000585097, K 000585098!, 00260319! US)!. *Coccoloba zernyi* Standl. in Publ. Field Mus. Nat. Hist., Bot. Ser. 22: 18 (1940). *Ruprechtia* zernyi (Standley) R. A. Howard, J. Arnold Arbor. 61: 390. 1960. —TYPE: Brazil. Amazonas: Taperinha, near Santarém, 13 Aug 1927, Zerny 890 (holotype: F934804!; isotype: GH 00055152 (photo & fragment)!.

##### Nomenclatural notes

The combination *Magoniella obidensis* (Huber) Adr.Sanchez, was published using *Ruprechtia obidensis* Huber, its older basionym (Sanchez and Kron, 2011). However, this author incorrectly cited an exsiccatae collected by Henry H. Rusby as the type specimen, the latter corresponded to a heterotypic synonym of *R. scandendes* Rusby from Bolivia (Mem. New York Bot. Gard. 7: 237. 1927). Therefore, the Rusby specimen does not correspond to the specimens cited by Huber in the protologue of *R. obidensis* (A. Ducke 2899, 2901). Whilst an erroneously cited specimen does not invalidate a combination (Turland et al., 2018, Art. 41.6), we here provide an exsiccatae correction through a basionym citation to avoid further ambiguity.

Huber (1909) described *Ruprechtia obidensis* using two specimens, Ducke 2899 ♀ and Ducke 2901 ♂; both were cited in the protologue. They did not designate either of the two as the holotype, so both specimens constitute syntypes according to Article 40, Note 1 of the International Code of Nomenclature for algae, fungi, and plants (ICN) (Turland et al., 2018). Pendry (2004) refers to the collection deposited in MG as the holotype specimen, with an isotype located in the BM herbarium. However, this cannot be considered an inadvertent lectotypification because the publication does not state that a lectotype is being designated and was published after 1 January 2001 (see Turland et al., 2018). Therefore, a lectotype still needs to be designated.

Subsequently, Pendry (2004) cited specimens collected by Ducke (8539 ♂ and 8540 ♀) stored at the herbarium BM, with isotypes in F, G, GH, MO, NY, as the holotype of *Ruprechtia macrocalyx.* However, when Huber (1909) described this species, they did not designate in the protologue any of the two specimens as the holotype. Again, according to Article 40, Note 1 of the ICN (Turland et al., 2018), both specimens are thus considered syntypes, and the designation of a lectotype is required. Because Pendry’s publication predates 1 January 2001, it also cannot be considered an inadvertent lectotypification. Therefore, the specimen Ducke 2899, ♀ (deposited in MG, accession 002899) and Ducke 8540, ♀ (deposited in MG, accession 008539) are selected as the lectotype of *R. obidensis* and *R. macrocalyx* respectively. These lectotypifications are done following Article 8.1 of the ICN (Turland et al., 2018), giving priority to the specimen that comes from the female plant since in this group the species diagnosis falls mostly on female reproductive characters. Additionally, we can check duplicates for both species at BM, F, and G.

#### 3. *Magoniella scandens* (Vell.) Aguilar-Cano **comb. nov**

**Basionym:** *Magonia scandens* Vell. in Fl. Flum.: 165. 1825. *Triplaris scandens* (Vell.) Cocucci, Revista Fac. Ci. Exact. 19: 361. 1957. *Magoniella laurifolia* (Cham. & Schltdl.) Adr. Sanchez, Syst. Bot. 36 (3): 708. 2011, comb. inval. nom. illeg.— TYPE: Vellozo’s illustration, Flora Fluminensis icones 4: t. 60 (lectotype designated by Pendry, 2004. *Triplaris laurifolia* Cham. & Schltdl, Linnaea 3: 55. 1828. *Ruprechtia laurifolia* (Cham. & Schltdl.) C. A. Meyer, Mém. Acad. Imp. Sei. Saint-Pétersbourg, Sér. 6, Sei. Math., Seconde Pt. Sei. Nat. 6: 150. 1840, *syn nov*. —TYPE: BRAZIL. “Brasilia aequinoctialis” Sello s.n. (lectotype designated by Pendry, 2004: B 101001716!; isolectotypes: B 101001717!, BR 5288069!, HAL 0098197)!.

##### Nomenclatural notes

*Magoniella laurifolia* (Cham. & Schltdl) Adr. Sanchez, is a combination proposed by Sanchez and Kron (2011) based on *Magonia scandens* Vell. (“Fl. Flum.: 165. 1825. — TYPE: Vellozo’s illustration, Flora Fluminensis icones 4: t. 60 [lectotype designated by Pendry, 2004]”) as the intended basionym. However, Sanchez proposed this combination based on *Triplaris laurifolia* Cham. & Schldl (in Sanchez & Kron, 2011; IPNI, 2022), therefore *M. laurifolia* is a name that is not validly published (comb. inval.), because Sanchez did not correctly cite the author of this basionym. The nomenclatural action of Sanchez represents an invalid combination due a mistake in the author’s name (*T. laurifolia* Cham. & Schldl., see: Turland et al., 2018, Art. 41.5). Additionally, according to Article 11.4 of the ICN, the epithet “*scandens*” is the earliest legitimate name available for a transfer to *Magoniella* even though it was published under *Magonia* Vell., a later homonym of *Magonia* A. St.-Hil. (Sapindaceae), Kuntze (1898) (Turland et al., 2018, Art. 55.1). Therefore, the new combination provided here under Article 6.10 (Turland et al., 2018), must replace *Magoniella laurifolia* (Cham. & Schltdl.) Adr. Sanchez. This name is nomenclaturally superfluous *sensu* the current taxonomy of *Magoniella* (Turland et al., 2018, Art. 52) because the name *M. laurifolia* included the type of a heterotypic synonym, whose epithet was available and not already pre-empted in the genus *Magoniella*. Hence, we here propose the new combination *Magoniella scandens* (Vell.) Aguilar-Cano based on *Magonia scandens* Vell.

### Key to the species of *Magoniella*

1. Pedicels shorter than the bracteoles, not noticeable after the ripe fruits have fallen……………………………………………………………………………………….**3. *M. scandens***.

1’. Pedicels longer than the tubular bracteole, clearly visible after the ripe fruits have fallen……………………………………………………………………………………………2.

2. Base of the perianth tube in the fruit abruptly taper to scars, resulting in a narrow, somewhat sharp base, completely covered with a dense indumentum; bracts 1.9–2.8 mm long……………………………………………………………………………………………….**1. *M. chersina***.

2’. Base of the perianth tube in the fruit no tapering to scar, resulting in a broad obtuse base, glabrescent to completely glabrous; bracts 1.3–2.8 mm long……….**2. *M. obidensis***.

## Data availability statement

The datasets presented in this study can be found in online repositories. The names of the repository/repositories and accession number(s) can be found in the article/**Supplementary Material**.

## Author contributions

JA-C and OAP-E conceived the study. JA-C produced the morphological diagnosis of the new species. OAP-E and JA-C produced the molecular diagnosis of the new species. JA-C developed the taxonomic treatment and conducted morphometrical analyses. JA-C and ET conducted wet-lab work. JA-C and OAP-E conducted molecular analyses. CP provided the pen and ink illustrations of this new species. JA-C prepared figures. JA-C and OAP-E wrote the manuscript, with contributions from CP, AA, and ET. All authors contributed to the article and approved the submitted version.

## Supplementary material

SUPPLEMENTARY FIGURE S1 Maximum likelihood phylograms inferred from combined **(A)** ITS + *lfy2i* nuclear loci and **(B)** combined *matK* + *ndhF* + *rps*16-*trnK* + *ndhC-trnV* plastid loci. Fully black pie charts at nodes indicate nodes attaining maximum statistical support. Circles preceding species names indicate potential outlier (red circle) and congruent (blue circle) terminals.

SUPPLEMENTARY FIGURE S2 Map Neotropical biomes of sampled species shown in front of tree terminals with coloured boxes preceding species names in Figure 1a. Based and adapted from terrestrial biomes proposed by Dinerstein et al. (2017).
