# Supplementary figures and images for: Isolated, neglected, and likely threatened: a new species of Magoniella (Polygonaceae) from the seasonally dry tropical forests of Northern Colombia and Venezuela revealed from nuclear, plastid, and morphological data
Source: Front Plant Sci. 2024 Jul 23;15:1253260. doi: 10.3389/fpls.2024.1253260 (PMC11301161; doi:10.3389/fpls.2024.1253260)

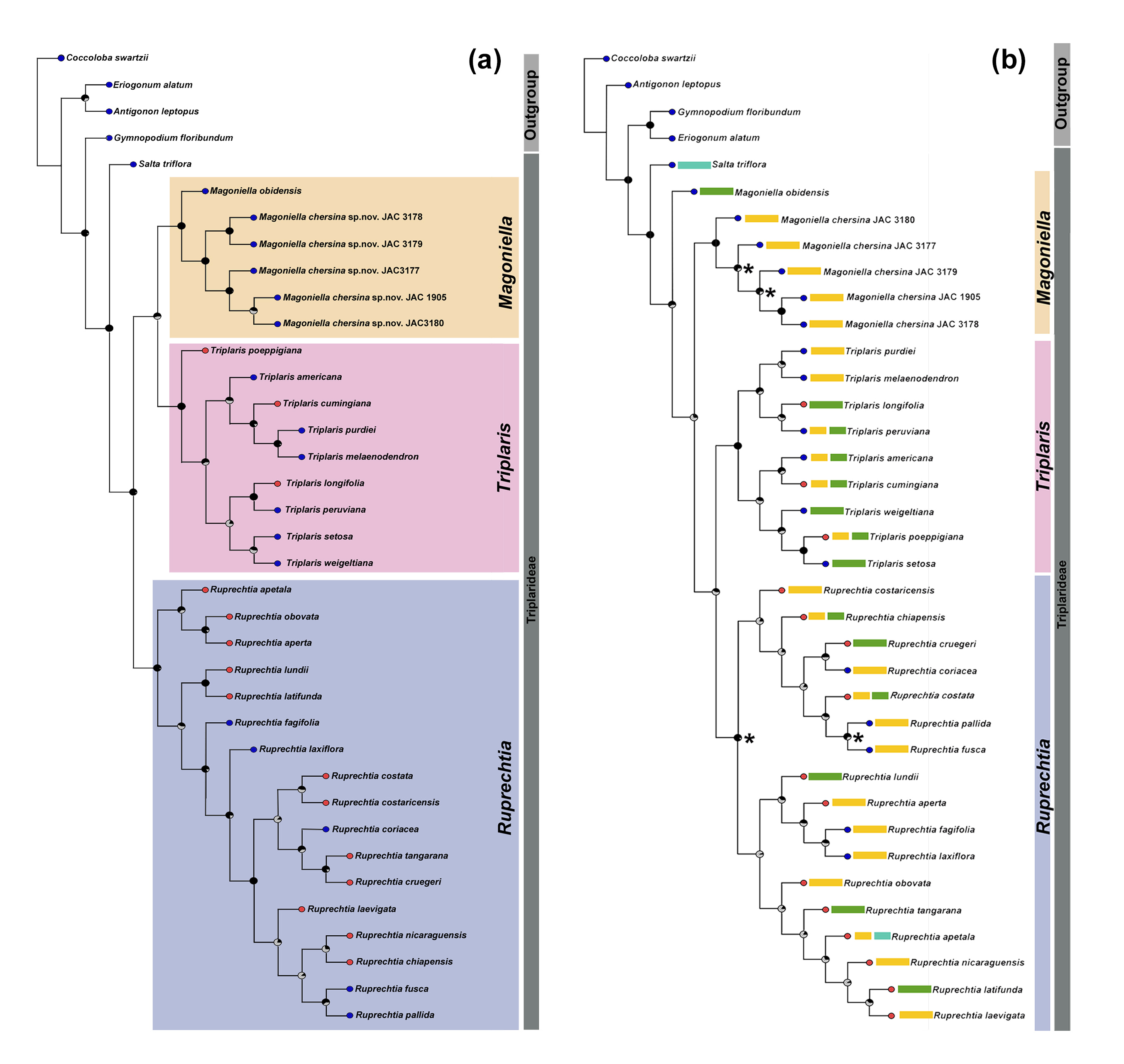

Supplement: SUPPLEMENTARY FIGURE S1 — Maximum likelihood phylograms inferred from combined (A) ITS + lfy2i nuclear loci and (B) combined matK + ndhF + rps16-trnK + ndhC-trnV plastid loci. Fully black pie charts at nodes indicate nodes attaining maximum statistical support. Circles preceding species names indicate potential outlier (red circle) and congruent (blue circle) terminals. [file Image_1.jpg]

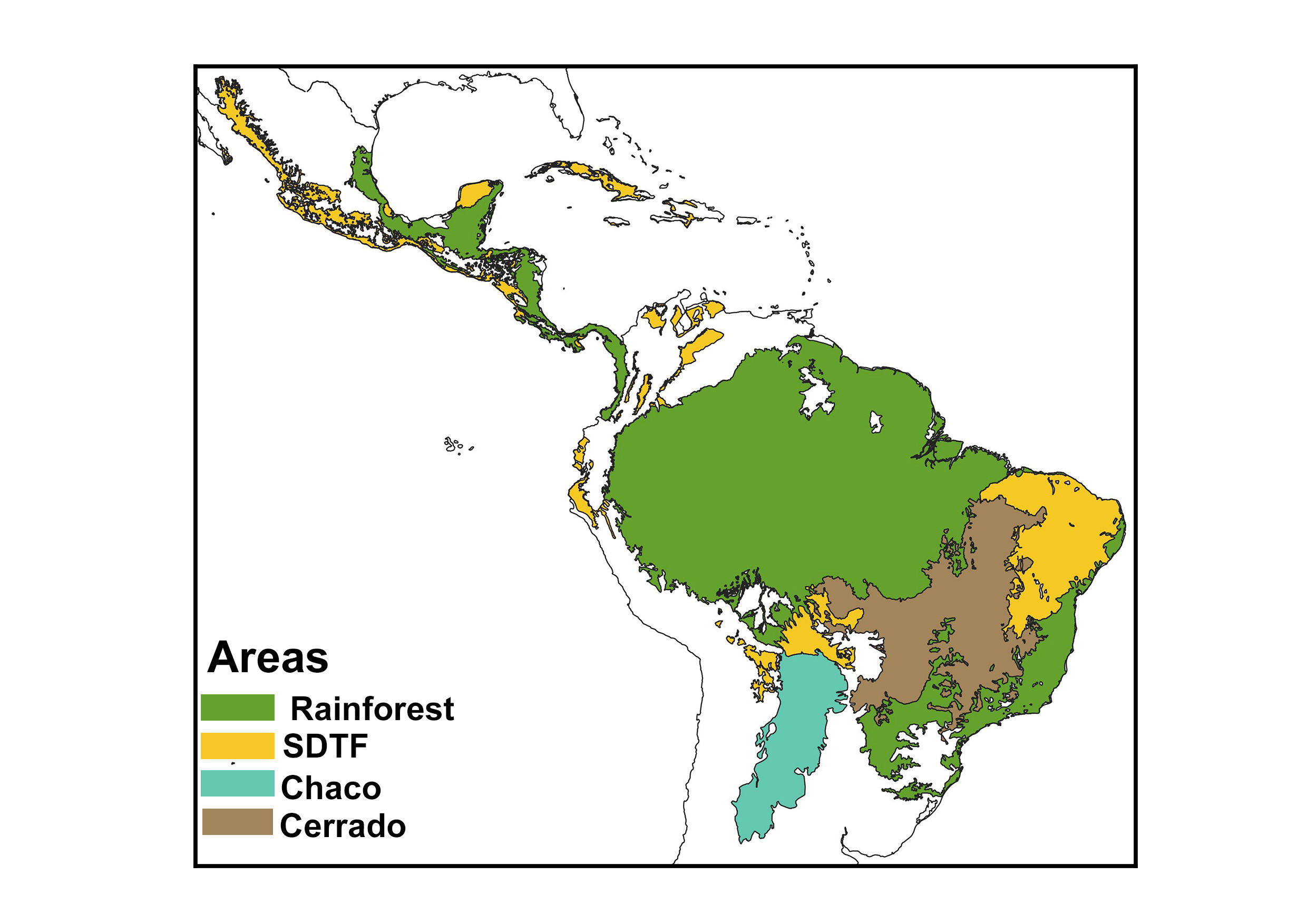

Supplement: SUPPLEMENTARY FIGURE S2 — Map Neotropical biomes of sampled species shown in front of tree terminals with coloured boxes preceding species names in Figure 1a. Based and adapted from terrestrial biomes proposed by Dinerstein et al. (2017). [file Image_2.jpg]
