# Supplementary material for: Isolated, neglected, and likely threatened: a new species of Magoniella (Polygonaceae) from the seasonally dry tropical forests of Northern Colombia and Venezuela revealed from nuclear, plastid, and morphological data
Source: Front Plant Sci. 2024 Jul 23;15:1253260. doi: 10.3389/fpls.2024.1253260 (PMC11301161; doi:10.3389/fpls.2024.1253260)
Supplement: Supplementary file 3 [file Table_1.docx]

***Supplementary Material***

**Misplaced, neglected, and likely threatened: A new species of *Magoniella* (Polygonaceae) from the seasonally dry tropical forests of Colombia and Venezuela revealed from nuclear, plastid and morphological data**

*** Correspondence:** Corresponding Authors: jose.aguilarcano@gmail.com or o.perez-escobar@kew.org

**Table S1**. Primers and PCR settings utilized for amplifying chloroplast and nuclear DNA loci in the present study.

| **DNA regions** | **Primer combination** | **Sequence** | **Reference** |
| --- | --- | --- | --- |
| rps16-trnK | rps16x2F2 | AAAGTGGGTTTTTATGATCC | (Shaw et al. 2007) |
|  | trnK (UUU) | TTAAAAGCCGAGTACTCTACC |  |
| ndhF | ndhF_1 | TGGAACAKACATATSAATATGC | (Olmstead & Sweere_1994) |
|  | ndhF_972R | CATCATATAACCCAATTGAGAC |  |
| ITS | ITS-5P | GGAAGGAGAAGTCGTAACAAGG | (Moller & Cronk_1997) |
|  | ITS-4 | TCCTCCGCTTATTGATATGC |  |
| lfy2i | lfy2i-3F | TGCAAGGGGTAAGAAGAACGGCCTTGA | (Sanchez & Kron 2011) |
|  | lfy2i-1R | CCTGCCGACATANTGGCGCATCTTGGGCTT |  |
