# Supplementary material for: Isolated, neglected, and likely threatened: a new species of Magoniella (Polygonaceae) from the seasonally dry tropical forests of Northern Colombia and Venezuela revealed from nuclear, plastid, and morphological data
Source: Front Plant Sci. 2024 Jul 23;15:1253260. doi: 10.3389/fpls.2024.1253260 (PMC11301161; doi:10.3389/fpls.2024.1253260)
Supplement: Supplementary file 4 [file Table_2.docx]

***Supplementary Material***

**Misplaced, neglected, and likely threatened: A new species of *Magoniella* (Polygonaceae) from the seasonally dry tropical forests of Colombia and Venezuela revealed from nuclear, plastid and morphological data**

*** Correspondence:** Corresponding Authors: jose.aguilarcano@gmail.com or o.perez-escobar@kew.org

**Table S2**. Information of species names and GenBank accession numbers obtained from GenBank and new sequences of *Magoniella chersina* generated and used in this study.

| **Species** | ***matK*** | ***ndhF*** | ***rps16-trnK*** | **ndhC-trnV** | **ITS** | ***lfy*2*i*** |
| --- | --- | --- | --- | --- | --- | --- |
| Antigonon_leptopus | EF437988 | EF438027 | HQ693194 | HQ693163 | FJ154462 | EF442788 |
| Coccoloba_swartzii | EF437995 | EF438034 | HQ693195 | HQ693164 | FJ154469 | EF442787 |
| Eriogonum_alatum | EF437998 | EF438038 | HQ693196 | HQ693165 | FJ154472 | EF438068 |
| Gymnopodium_floribundum | GQ206197 | GQ206282 | HQ693197 | HQ693166 | GQ206251 | HQ693138 |
| Magoniella_obidensis | HQ693198 | HQ693214 | HQ693167 | HQ693151 | HQ693103 | HQ693137 |
| Magoniella chersina_Aguilar-Cano 1905 | - | OM990664 | OM990669 | - | OM990654 | OM990659 |
| Magoniella chersina_Aguilar-Cano-3177 | - | OM990665 | OM990670 | - | OM990655 | OM990660 |
| Magoniella chersina_Aguilar-Cano-3178 | - | OM990666 | OM990671 | - | OM990656 | OM990661 |
| Magoniella chersina_Aguilar-Cano-3179 | - | OM990667 | OM990672 | - | OM990657 | OM990662 |
| Magoniella chersina_Aguilar-Cano-3180 | - | OM990668 | OM990673 | - | OM990658 | OM990663 |
| Ruprechtia_aperta | HQ693199 | HQ693215 | HQ693169 | HQ693139 | HQ693104 | HQ693118 |
| Ruprechtia_apetala | HQ693200 | HQ693216 | HQ693170 | HQ693140 | HQ693105 | - |
| Ruprechtia_chiapensis | FJ154495 | FJ154506 | HQ693171 | HQ693141 | FJ154482 | HQ693119 |
| Ruprechtia_coriacea | HM137401 | HM137423 | HQ693172 | HQ693142 | HM137442 | HQ693120 |
| Ruprechtia_costaricensis | HQ693201 | HQ693217 | HQ693173 | HQ693143 | HQ693106 | HQ693121 |
| Ruprechtia_costata | HQ693202 | HQ693218 | HQ693174 | - | HQ693107 | - |
| Ruprechtia_cruegeri | HM137402 | HM137424 | HQ693175 | HQ693144 | HM137443 | - |
| Ruprechtia_fagifolia | HQ693203 | HQ693219 | HQ693176 | HQ693145 | HQ693108 | - |
| Ruprechtia_fusca | FJ154496 | FJ154507 | HQ693177 | HQ693146 | FJ154483 | HQ693122 |
| Ruprechtia_laevigata | HQ693204 | HQ693220 | HQ693178 | HQ693147 | HQ693109 | - |
| Ruprechtia_latifunda | HQ693205 | - | - | - | HQ693110 | HQ693123 |
| Ruprechtia_laxiflora | EF438024 | EF438063 | HQ693179 | HQ693148 | FJ154484 | HQ693124 |
| Ruprechtia_lundii | HQ693206 | HQ693221 | HQ693180 | HQ693149 | HQ693111 | HQ693125 |
| Ruprechtia_nicaraguensis | HQ693207 | HQ693222 | HQ693181 | HQ693150 | HQ693112 | HQ693126 |
| Ruprechtia_obovata | HQ693208 | HQ693223 | HQ693182 | HQ693152 | HQ693113 | HQ693127 |
| Ruprechtia_pallida | HM137403 | HM137425 | HQ693183 | - | HM137445 | - |
| Ruprechtia_tangarana | EF438025 | EF438064 | HQ693184 | HQ693153 | FJ154485 | HQ693128 |
| Salta_triflora | HQ693213 | GQ206299 | HQ693168 | HQ693154 | GQ206267 | - |
| Triplaris_americana | AY042668 | FJ154508 | HQ693185 | HQ693155 | FJ154486 | HQ693129 |
| Triplaris_cumingiana | GQ206210 | GQ206301 | HQ693186 | HQ693156 | GQ206269 | HQ693130 |
| Triplaris_longifolia | HQ693209 | HQ693224 | HQ693187 | - | HQ693114 | HQ693131 |
| Triplaris_melaenodendron | HQ693210 | HQ693225 | HQ693188 | HQ693157 | HQ693115 | HQ693132 |
| Triplaris_peruviana | HQ693211 | HQ693226 | HQ693189 | HQ693158 | HQ693116 | HQ693133 |
| Triplaris_poeppigiana | FJ154497 | FJ154509 | HQ693190 | HQ693159 | FJ154487 | HQ693134 |
| Triplaris_purdiei | HQ693212 | HQ693227 | HQ693191 | HQ693160 | HQ693117 | - |
| Triplaris_setosa | FJ154498 | FJ154510 | HQ693192 | HQ693161 | FJ154488 | HQ693135 |
| Triplaris_weigeltiana | HM137405 | HM137426 | HQ693193 | HQ693162 | HM137446 | HQ693136. |
