# Supplementary material for: Isolated, neglected, and likely threatened: a new species of Magoniella (Polygonaceae) from the seasonally dry tropical forests of Northern Colombia and Venezuela revealed from nuclear, plastid, and morphological data
Source: Front Plant Sci. 2024 Jul 23;15:1253260. doi: 10.3389/fpls.2024.1253260 (PMC11301161; doi:10.3389/fpls.2024.1253260)
Supplement: Supplementary file 5 [file Table_3.docx]

***Supplementary Material***

**Misplaced, neglected, and likely threatened: A new species of *Magoniella* (Polygonaceae) from the seasonally dry tropical forests of Colombia and Venezuela revealed from nuclear, plastid and morphological data**

*** Correspondence:** Corresponding Authors: jose.aguilarcano@gmail.com or o.perez-escobar@kew.org

**Table S3**. Species names, voucher information and herbarium specimens of

voucher housed for material used in this study to build morphometric matrix of Two-Sample Fisher-Pitman Permutation Test.

| **Species** | **Voucher** | **Herbarium** | **Ripe fruit length** | **Perianth tube length** | **Perianth tube width** | **Sepal of fruits lenght** | **Sepal of fruits width** | **Bracteoles length** |
| --- | --- | --- | --- | --- | --- | --- | --- | --- |
| *Magoniella chersina* | Aguilar-1905 | FMB | 33,206 | 7,976 | 3,849 | 25,026 | 5,559 | 2,463 |
| *Magoniella chersina* | Aguilar-1905 | FMB | 40,18 | 9,928 | 3,865 | 29,673 | 6,303 | 2,577 |
| *Magoniella chersina* | Aguilar-1905 | FMB | 32,847 | 8,494 | 3,996 | 24,272 | 5,5 | 2,503 |
| *Magoniella chersina* | Allen-929 | K | 45,206 | 12,113 | 3,71 | 33,138 | 6,929 | 2,038 |
| *Magoniella chersina* | Allen-929 | K | 43,957 | 12,437 | 4,063 | 30,475 | 5,676 | 1,904 |
| *Magoniella chersina* | Allen-929 | K | 44,543 | 11,103 | 4,453 | 32,849 | 6,426 | 2,127 |
| *Magoniella chersina* | Pittier-13302 | MO | 22,574 | 9,428 | 4,125 | 13,411 | 3,218 | 2,458 |
| *Magoniella chersina* | Pittier-13302 | MO | 23,831 | 9,573 | 4,169 | 14,096 | 3,379 | 2,454 |
| *Magoniella chersina* | Pittier-13302 | MO | 25,196 | 7,361 | 4,181 | 16,816 | 4,179 | 2,619 |
| *Magoniella chersina* | Pittier-13302 | MO | 28,799 | 9,586 | 4,364 | 17,708 | 4,426 | 2,829 |
| *Magoniella laurifolia* | Duarte-932 | RB | 28,45 | 8,064 | 4,243 | 20,9 | 6,515 | - |
| *Magoniella laurifolia* | Duarte-932 | RB | 32,817 | 11,257 | 4,776 | 20,897 | 6,151 | - |
| *Magoniella laurifolia* | Duarte-932 | RB | 31,67 | 9,283 | 4,934 | 21,734 | 7,12 | - |
| *Magoniella laurifolia* | Fonseca-945 | RB | 45,651 | 8,624 | 4,595 | 36,578 | 7,362 | 2,704 |
| *Magoniella laurifolia* | Fonseca-945 | RB | 45,388 | 9,103 | 5,049 | 35,411 | 8,461 | 2,553 |
| *Magoniella laurifolia* | Fonseca-945 | RB | 47,35 | 9,832 | 5,714 | 37,08 | 8,498 | 2,811 |
| *Magoniella laurifolia* | Leoni-4000a | RB | 32,056 | 10,043 | 4,573 | 22,116 | 5,481 | 3,971 |
| *Magoniella laurifolia* | Leoni-4000a | RB | 29,318 | 9,99 | 5,389 | 18,956 | 4,899 | 3,709 |
| *Magoniella laurifolia* | Leoni-4000a | RB | 33,14 | 8,083 | 5,469 | 25,251 | 5,768 | 3,773 |
| *Magoniella laurifolia* | Leoni-4000b | RB | 27,889 | 6,763 | 4,523 | 21,027 | 4,466 | 3,87 |
| *Magoniella laurifolia* | Leoni-4000b | RB | 26,682 | 9,085 | 5,059 | 17,215 | 3,805 | 3,555 |
| *Magoniella laurifolia* | Leoni-4000b | RB | 23,459 | 7,146 | 5,071 | 16,365 | 3,616 | 3,94 |
| *Magoniella laurifolia* | Marquete-634 | RB | 28,995 | 8,29 | 4,345 | 19,75 | 4,745 | 2,958 |
| *Magoniella laurifolia* | Marquete-634 | RB | 27,138 | 7,392 | 4,353 | 20,528 | 4,775 | 2,876 |
| *Magoniella laurifolia* | Marquete-634 | RB | 25,179 | 8,619 | 4,936 | 17,438 | 3,631 | 2,873 |
| *Magoniella laurifolia* | Marquete-634 | K | 42,854 | 8,432 | 5,842 | 34,376 | 7,898 | 2,851 |
| *Magoniella laurifolia* | Marquete-634 | K | 38,182 | 10,497 | 5,869 | 29,847 | 7,131 | 2,674 |
| *Magoniella laurifolia* | Marquete-634 | K | 45,255 | 9,191 | 6,379 | 36,004 | 9,027 | 2,692 |
| *Magoniella laurifolia* | Petroa 6703 | K | 32,954 | 11,937 | 4,645 | 20,837 | 4,248 | 3,077 |
| *Magoniella laurifolia* | Petroa 6703 | K | 39,944 | 12,462 | 5,303 | 27,302 | 5,444 | 2,774 |
| *Magoniella laurifolia* | Petroa 6703 | K | 42,04 | 13,28 | 5,67 | 27,926 | 6,165 | 2,673 |
| *Magoniella laurifolia* | Sellow-s.n | HAL | 19,72 | 8,09 | 3,13 | 12,41 | 2,92 | 2,88 |
| *Magoniella laurifolia* | Sellow-s.n. | B | 22,68 | 6,89 | 3,09 | 15,23 | 3,75 | 4,39 |
| *Magoniella laurifolia* | Sellow-s.n. | B | 23,59 | 9,14 | 3,43 | 15,14 | 3,48 | 4,17 |
| *Magoniella laurifolia* | Sellow-s.n. | B-HV | 22,144 | 10,202 | 3,518 | 12,232 | 3,296 | 4,091 |
| *Magoniella laurifolia* | Sellow-s.n. | HAL | 23,39 | 10,57 | 3,54 | 13,66 | 3,95 | 2,97 |
| *Magoniella laurifolia* | Sellow-s.n. | B-HV | 24,123 | 8,685 | 3,634 | 16,833 | 3,323 | 4,139 |
| *Magoniella laurifolia* | Sellow-s.n. | B-HV | 22,64 | 8,573 | 3,714 | 14,041 | 2,907 | 3,649 |
| *Magoniella laurifolia* | Sellow-s.n. | B | 26,21 | 7,98 | 3,79 | 16,86 | 3,69 | 4,26 |
| *Magoniella laurifolia* | Sucre-3512 | RB | 34,237 | 9,3 | 3,916 | 25,078 | 7,069 | 3,926 |
| *Magoniella laurifolia* | Sucre-3512 | RB | 29,097 | 9,745 | 4,099 | 19,476 | 5,762 | 3,596 |
| *Magoniella laurifolia* | Sucre-3512 | RB | 36,076 | 9,643 | 4,867 | 26,305 | 7,799 | 3,617 |
| *Magoniella laurifolia* | Vasconcelos-s.n. | HUEFS | 25,03 | 11,086 | 3,742 | 13,716 | 3,149 | 3,327 |
| *Magoniella laurifolia* | Vasconcelos-s.n. | HUEFS | 27,253 | 11,413 | 3,857 | 15,942 | 3,787 | 3,589 |
| *Magoniella laurifolia* | Vasconcelos-s.n. | HUEFS | 29,777 | 12,91 | 4,358 | 16,615 | 4,556 | 3,805 |
| *Magoniella laurifolia* | Sucre-3512 | K | 34,736 | 10,266 | 4,518 | 24,924 | 7,659 | 3.822 |
| *Magoniella laurifolia* | Sucre-3512 | K | 36,12 | 9,606 | 4,701 | 26,36 | 6,444 | 3.954 |
| *Magoniella laurifolia* | Sucre-3512 | K | 45,893 | 15,868 | 6,774 | 31,075 | 8,722 | 4,25 |
| *Magoniella obidensis* | Baldwin Jr.-2751 | US | 49,637 | 12,188 | 4,699 | 37,717 | 10,408 | 1,962 |
| *Magoniella obidensis* | Baldwin Jr.-2751 | US | 46,241 | 10,63 | 4,757 | 35,884 | 9,691 | 1,961 |
| *Magoniella obidensis* | Baldwin Jr.-2751 | IAN | 48,341 | 15,473 | 4,815 | 31,824 | 8,613 | 1,678 |
| *Magoniella obidensis* | Baldwin Jr.-2751 | IAN | 47,432 | 11,955 | 4,853 | 35,431 | 9,581 | 1,614 |
| *Magoniella obidensis* | Baldwin Jr.-2751 | US | 48,961 | 12,993 | 4,882 | 35,256 | 8,609 | 1,965 |
| *Magoniella obidensis* | Baldwin Jr.-2751 | IAN | 57,338 | 19,87 | 7,325 | 38,739 | 11,196 | 1,533 |
| *Magoniella obidensis* | Ducke 19542 | K | 39,12 | 8,942 | 5,491 | 31,27 | 10,017 | 1,412 |
| *Magoniella obidensis* | Ducke 19542 | K | 44,349 | 8,231 | 5,802 | 36,185 | 10,127 | 1,53 |
| *Magoniella obidensis* | Ducke 19542 | K | 43,481 | 9,169 | 6,7 | 34,772 | 9,345 | 1,408 |
| *Magoniella obidensis* | Ducke-19545 | RB | 40,176 | 8,274 | 5,682 | 33,014 | 8,085 | 1,617 |
| *Magoniella obidensis* | Ducke-19545 | RB | 38,909 | 13,54 | 6,56 | 25,46 | 6,486 | 1,661 |
| *Magoniella obidensis* | Ducke-2899 | MG | 41,312 | 12,625 | 4,106 | 27,39 | 3,929 | 1,834 |
| *Magoniella obidensis* | Ducke-2899 | MG | 37,311 | 14,106 | 4,377 | 23,141 | 4,203 | 1,872 |
| *Magoniella obidensis* | Ducke-2899 | MG | 39,262 | 12,814 | 4,445 | 26,075 | 4,089 | 1,758 |
| *Magoniella obidensis* | Ducke-2899 | F | 46,88 | 13,45 | 5,68 | 32,57 | 5,01 | 1,72 |
| *Magoniella obidensis* | Ducke-8540 | BM | 49,01 | 14,05 | 6,67 | 37,75 | 9,94 | 1,66 |
| *Magoniella obidensis* | Ducke-8540 | BM | 49,94 | 13,19 | 6,87 | 35,85 | 10,79 | 1,87 |
| *Magoniella obidensis* | Ginzberger-916 | W | 54,54 | 12,055 | 4,723 | 42,251 | 5,881 | 1,34 |
| *Magoniella obidensis* | Ginzberger-916 | W | 43,953 | 11,299 | 4,828 | 33,848 | 6,793 | 1,535 |
| *Magoniella obidensis* | Ginzberger-916 | W | 51,711 | 12,401 | 5,107 | 38,781 | 7,66 | 1,574 |
| *Magoniella obidensis* | Lowrie-386 | NY | 35,144 | 7,828 | 4,477 | 27,544 | 8,932 | 1,31 |
| *Magoniella obidensis* | Lowrie-386 | NY | 34,084 | 8,632 | 5,348 | 25,601 | 8,916 | 1,71 |
| *Magoniella obidensis* | Prance-6991 | NY | 45,781 | 11,627 | 7,34 | 36,891 | 9,372 | - |
| *Magoniella obidensis* | Prance-6991 | K | 47,189 | 9,59 | 7,656 | 37,731 | 8,719 | 1,84 |
| *Magoniella obidensis* | Prance-6991 | K | 43,13 | 9,7 | 7,736 | 33,286 | 9,346 | 1,902 |
| *Magoniella obidensis* | Prance-6991 | K | 46,517 | 9,658 | 7,889 | 36,396 | 9,128 | 1,875 |
