# Supplementary material for: Isolated, neglected, and likely threatened: a new species of Magoniella (Polygonaceae) from the seasonally dry tropical forests of Northern Colombia and Venezuela revealed from nuclear, plastid, and morphological data
Source: Front Plant Sci. 2024 Jul 23;15:1253260. doi: 10.3389/fpls.2024.1253260 (PMC11301161; doi:10.3389/fpls.2024.1253260)
Supplement: Supplementary file 6 [file Table_4.docx]

***Supplementary Material***

**Misplaced, neglected, and likely threatened: A new species of *Magoniella* (Polygonaceae) from the seasonally dry tropical forests of Colombia and Venezuela revealed from nuclear, plastid and morphological data**

*** Correspondence:** Corresponding Authors: jose.aguilarcano@gmail.com or o.perez-escobar@kew.org

**Table S4**. Presence/absence matrix of Triplarideae species to define their geographic distribution in the major Neotropical biomes shown in Figure 1b. Adapted from terrestrial biomes proposed by Dinerstein et al. (2017).

| **Species** | **Major Neotropical biomes** | | | | | | | | | | | | | | |  |
| --- | --- | --- | --- | --- | --- | --- | --- | --- | --- | --- | --- | --- | --- | --- | --- | --- |
|  | STDF-Caatingas | STDF-Misiones Nucleus | STDF-Caribbean coast COL & VEN | STDF-Pacific coastal ECU & PER | STDF-N PER Inter-Andean | STDF-COL & VEN Inter-Andean | STDF-Mexico and Central America | STDF-Bolivian piedmont nucleus | STDF-Caribbean Islands | Chaco | Cerrado | Rainforest Amazon-Guinas | Central American rainforest | Eastern Brazilian Atlantic coastal rainforest | Rainforest Choco | Total records |
| ***Magoniella_obidensis*** | 0 | 0 | 0 | 0 | 0 | 0 | 0 | 0 | 0 | 0 | 0 | 1 | 0 | 0 | 0 | 2 |
| ***Magoniella chersina* sp. nov.** | 0 | 0 | 1 | 0 | 0 | 0 | 0 | 0 | 0 | 0 | 0 | 0 | 0 | 0 | 0 | 1 |
| ***Ruprechtia_aperta*** | 0 | 0 | 0 | 1 | 0 | 0 | 0 | 0 | 0 | 0 | 0 | 0 | 0 | 0 | 0 | 1 |
| ***Ruprechtia_apetala*** | 0 | 0 | 0 | 0 | 0 | 0 | 0 | 1 | 0 | 1 | 0 | 0 | 0 | 0 | 0 | 2 |
| ***Ruprechtia_chiapensis*** | 0 | 0 | 0 | 0 | 0 | 0 | 1 | 0 | 0 | 0 | 0 | 0 | 1 | 0 | 0 | 2 |
| ***Ruprechtia_coriacea*** | 0 | 0 | 1 | 0 | 0 | 0 | 0 | 0 | 0 | 0 | 0 | 0 | 0 | 0 | 0 | 1 |
| ***Ruprechtia_costaricensis*** | 0 | 0 | 0 | 0 | 0 | 0 | 1 | 0 | 0 | 0 | 0 | 0 | 0 | 0 | 0 | 1 |
| ***Ruprechtia_costata*** | 0 | 0 | 0 | 0 | 0 | 0 | 1 | 0 | 0 | 0 | 0 | 0 | 1 | 0 | 0 | 2 |
| ***Ruprechtia_cruegeri*** | 0 | 0 | 0 | 0 | 0 | 0 | 0 | 0 | 0 | 0 | 0 | 1 | 0 | 0 | 0 | 1 |
| ***Ruprechtia_fagifolia*** | 1 | 0 | 0 | 0 | 0 | 0 | 0 | 0 | 0 | 0 | 0 | 0 | 0 | 0 | 0 | 1 |
| ***Ruprechtia_fusca*** | 0 | 0 | 0 | 0 | 0 | 0 | 1 | 0 | 0 | 0 | 0 | 0 | 0 | 0 | 0 | 1 |
| ***Ruprechtia_laevigata*** | 0 | 0 | 0 | 0 | 0 | 0 | 1 | 0 | 0 | 0 | 0 | 0 | 0 | 0 | 0 | 1 |
| ***Ruprechtia_latifunda*** | 0 | 0 | 0 | 0 | 0 | 0 | 0 | 0 | 0 | 0 | 0 | 0 | 0 | 1 | 0 | 1 |
| ***Ruprechtia_laxiflora*** | 1 | 1 | 0 | 0 | 0 | 0 | 0 | 1 | 0 | 0 | 0 | 0 | 0 | 0 | 0 | 3 |
| ***Ruprechtia_lundii*** | 0 | 0 | 0 | 0 | 0 | 0 | 0 | 0 | 0 | 0 | 0 | 0 | 0 | 1 | 0 | 1 |
| ***Ruprechtia_nicaraguensis*** | 0 | 0 | 0 | 0 | 0 | 0 | 1 | 0 | 0 | 0 | 0 | 0 | 0 | 0 | 0 | 1 |
| ***Ruprechtia_obovata*** | 0 | 0 | 0 | 0 | 1 | 0 | 0 | 0 | 0 | 0 | 0 | 0 | 0 | 0 | 0 | 1 |
| ***Ruprechtia_pallida*** | 0 | 0 | 0 | 0 | 0 | 0 | 1 | 0 | 0 | 0 | 0 | 0 | 0 | 0 | 0 | 1 |
| ***Ruprechtia_tangarana*** | 0 | 0 | 0 | 0 | 0 | 0 | 0 | 0 | 0 | 0 | 0 | 1 | 0 | 0 | 0 | 1 |
| ***Salta_triflora*** | 0 | 0 | 0 | 0 | 0 | 0 | 0 | 0 | 0 | 1 | 0 | 0 | 0 | 0 | 0 | 1 |
| ***Triplaris_americana*** | 0 | 0 | 1 | 0 | 1 | 1 | 0 | 0 | 0 | 0 | 0 | 1 | 0 | 0 | 1 | 5 |
| ***Triplaris_cumingiana*** | 0 | 0 | 1 | 1 | 0 | 1 | 0 | 0 | 0 | 0 | 0 | 1 | 1 | 0 | 1 | 6 |
| ***Triplaris_longifolia*** | 0 | 0 | 0 | 0 | 0 | 0 | 0 | 0 | 0 | 0 | 0 | 1 | 0 | 0 | 0 | 1 |
| ***Triplaris_melaenodendron*** | 0 | 0 | 0 | 0 | 0 | 1 | 1 | 0 | 0 | 0 | 0 | 0 | 0 | 0 | 0 | 2 |
| ***Triplaris_peruviana*** | 0 | 0 | 0 | 0 | 1 | 0 | 0 | 0 | 0 | 0 | 0 | 1 | 0 | 0 | 0 | 2 |
| ***Triplaris_poeppigiana*** | 0 | 0 | 0 | 0 | 1 | 0 | 0 | 0 | 0 | 0 | 0 | 1 | 0 | 0 | 0 | 2 |
| ***Triplaris_purdiei*** | 0 | 0 | 1 | 0 | 0 | 0 | 0 | 0 | 0 | 0 | 0 | 0 | 0 | 0 | 0 | 1 |
| ***Triplaris_setosa*** | 0 | 0 | 0 | 0 | 0 | 0 | 0 | 0 | 0 | 0 | 0 | 1 | 0 | 0 | 0 | 1 |
| ***Triplaris_weigeltiana*** | 0 | 0 | 0 | 0 | 0 | 0 | 0 | 0 | 0 | 0 | 0 | 1 | 0 | 0 | 0 | 1 |
