# Supplementary material for: Isolated, neglected, and likely threatened: a new species of Magoniella (Polygonaceae) from the seasonally dry tropical forests of Northern Colombia and Venezuela revealed from nuclear, plastid, and morphological data
Source: Front Plant Sci. 2024 Jul 23;15:1253260. doi: 10.3389/fpls.2024.1253260 (PMC11301161; doi:10.3389/fpls.2024.1253260)
Supplement: Supplementary file 7 [file Table_5.docx]

***Supplementary Material***

**Misplaced, neglected, and likely threatened: A new species of *Magoniella* (Polygonaceae) from the seasonally dry tropical forests of Colombia and Venezuela revealed from nuclear, plastid and morphological data**

*** Correspondence:** Corresponding Authors: jose.aguilarcano@gmail.com or o.perez-escobar@kew.org

**Table S5**. Results of Two-Sample Fisher-Pitman Permutation Test Differences comparing six morphological characters of ripe fruits among species of *Magoniella*. 95% confidence intervals in parentheses: *M. chersina* (8,6021-10,998 mm), *M. laurifolia* (9,0453-10,288 mm), *M. obidensis* (10,614-12,793 mm).

|  | Magoniella laurifolia | Magoniella obidensis |
| --- | --- | --- |
|  | **Ripe fruit length** | |
| Magoniella chersina | 0,44552 | **0,00010674** |
| Magoniella laurifolia |  | 6,6896x10-10 |
|  | **Perianth tube length** | |
| Magoniella chersina | 0,84058 | **0,045798** |
| Magoniella laurifolia |  | **0,00072951** |
|  | **Perianth tube width** | |
| Magoniella chersina | 0,064966 | **0,00014289** |
| Magoniella laurifolia |  | **7,2077x10-5** |
|  | **Sepal of fruits lenght** | |
| Magoniella chersina | 0,55546 | **7,1854x10-5** |
| Magoniella laurifolia |  | **2,9598x10-9** |
|  | **Sepal of fruits width** | |
| Magoniella chersina | 0,63596 | **0,00013634** |
| Magoniella laurifolia |  | **5,8346x10-7** |
|  | **Bracteoles length** | |
| Magoniella chersina | **1,6134x10-6** | **9,5101x10-10** |
| Magoniella laurifolia |  | **3,4466x10-21** |
